# Proteomic analysis revealed common, unique and systemic signatures in gender-dependent hepatocarcinogenesis

**DOI:** 10.1186/s13293-020-00316-5

**Published:** 2020-08-13

**Authors:** Huiling Li, Zhuona Rong, Hong Wang, Nan Zhang, Chunwen Pu, Yi Zhao, Xu Zheng, Chuanyi Lei, Yang Liu, Xiaoqin Luo, Jun Chen, Fujin Wang, Aiguo Wang, Jingyu Wang

**Affiliations:** 1 Department of Comparative Medicine, Laboratory Animal Center, Dalian Medical University, Dalian, 116044 Liaoning China; 2 Key Laboratory of Carcinogenesis and Translational Research (Ministry of Education/Beijing), Department of Biochemistry and Molecular Biology, Peking University Cancer Hospital & Institute, No. 52 Fucheng Road, Beijing, 100142 China; 3 Department of Biobank, The Affiliated Sixth People’s Hospital of Dalian Medical University, Dalian, 116031 China

**Keywords:** Hepatocellular carcinoma, Gender disparity, *Ras* oncogene, Proteomics, Tandem-mass-tag (TMT)

## Abstract

Hepatocellular carcinoma (HCC) is the most common liver cancer and is highly malignant. Male prevalence and frequent activation of the Ras signaling pathway are distinct characteristics of HCC. However, the underlying mechanisms remain to be elucidated. By exploring *Hras12V* transgenic mice showing male-biased hepatocarcinogenesis, we performed a high-throughput comparative proteomic analysis based on tandem-mass-tag (TMT) labeling combined with liquid chromatography-tandem mass spectrometry (LC-MS/MS) on the tissue samples obtained from HCC (T) and their paired adjacent precancerous (P) of *Hras12V* transgenic male and female mice (Ras-Tg) and normal liver (W) of wild-type male and female mice (Non-Tg). The further validation and investigation were performed using quantitative real-time PCR and western blot. Totally, 5193 proteins were quantified, originating from 5733 identified proteins. Finally, 1344 differentially expressed proteins (DEPs) (quantified in all examined samples; |ratios| ≥ 1.5, *p* < 0.05) were selected for further analysis. Comparison within W, P, and T of males and females indicated that the number of DEPs in males was much higher than that in females. Bioinformatics analyses showed the common and unique cluster-enriched items between sexes, indicating the common and gender-disparate pathways towards HCC. Expression change pattern analysis revealed HCC positive/negative-correlated and *ras* oncogene positive/negative-correlated DEPs and pathways. In addition, it showed that the *ras* oncogene gradually and significantly reduced the responses to sex hormones from hepatocytes to hepatoma cells and therefore shrunk the gender disparity between males and females, which may contribute to the cause of the loss of HCC clinical responses to the therapeutic approaches targeting sex hormone pathways. Additionally, gender disparity in the expression levels of key enzymes involved in retinol metabolism and terpenoid backbone/steroid biosynthesis pathways may contribute to male prevalence in hepatocarcinogenesis. Further, the biomarkers, SAA2, Orm2, and Serpina1e, may be sex differences. In conclusion, common and unique DEPs and pathways toward HCC initiated by *ras* oncogene from sexually dimorphic hepatocytes provide valuable and novel insights into clinical investigation and practice.

## Introduction

HCC is the most common primary liver cancer and one of the severest malignancies dangerous for human’s health. A universal feature of HCC in almost all populations is a notable male prevalence regardless of etiologies such as infections with hepatitis B (HBV) and hepatitis C (HCV) virus, alcohol-induced liver injury, environmental toxins, and nonalcoholic steatohepatitis [1, 2]. Interestingly, animal studies also showed that male rodents are more susceptible to hepatocarcinogenesis that occurs either spontaneously or is chemically and oncogenetically induced as well as in experimental models of chronic viral infection [3–5]. This indicates that there is a gender-dependent regulation of common molecular mechanisms leading to a predominance of HCC occurring in male humans and rodents [4]. Therefore, dissecting the underlying mechanisms responsible for this gender disparity using animal models will shed light on clinical investigations.

Ras plays crucial roles in controlling cell proliferation and differentiation [6]. Many studies have shown that the mutational activation of Ras and Ras-related signaling pathways play vital roles during the occurrence and development of HCC [7]. As an important member of the Ras family, H*ras* mutation has been widely investigated in multiple types of solid tumors, and H*ras* mutation may play an important role in the genesis and development of HCC [8]. We have established a transgenic mouse expressing the liver-specific *Hras12V* oncogene that develops liver cancer at the appropriate time and with high incidence in males but not in females [4]. It is suitable for exploring the mechanisms about the *ras* oncogene-induced HCC and the male-biased hepatocarcinogenesis.

Proteomics analysis is increasingly employed in global evaluation of protein expression [9], and multiple proteomic approaches have been used to study various aspects of liver tumor researches, such as screening molecular biomarkers for early diagnosis and prediction and investigating the underlying molecular mechanisms. With the development of new proteomics strategies, especially stable isotope labeling and multi-dimensional protein identification technology, it is now possible to track the changes of a thousand physiologically relevant proteins within a sample containing no more than 100 μg of protein [10]. It enables us to further explore the molecular events in HCC induced by *ras* oncogene and male prevalence. Mass spectrometry-based proteomics is a revolutionary technology that can rapidly identify and accurately quantify thousands of proteins within a complex biological specimen [10].

In this research, we performed high-throughput quantitative proteomic analysis in tissues obtained from HCC and adjacent precancerous of *Hras12V* transgenic mice and normal liver of wild-type mice ( 9 months old males and 15 months old females) by using TMT labeling coupled with LC-MS/MS. Totally, 5193 proteins were quantified, originating from 5733 identified proteins. Subsequently, bioinformatics analysis and biological validation cast new lights on our understanding of the underlying intracellular mechanisms in male prevalence and *ras* oncogene-related hepatocarcinogenesis.

## Materials and methods

### Animals, sampling, and histopathological diagnosis

Procedures for animal handling and tissue sampling were conducted in compliance with protocols approved by the Animal Care and Use Committee of Dalian Medical University. All procedures used in the study for animal breeding, handling, and sampling were performed as described in our previously published paper [11]. Briefly, after sacrificing mice by cervical dislocation, sampled tissues were washed in ice-cold sterile PBS to remove contaminating blood and then cut into 1 mm^3^ pieces using tissue scissors. Immediately, parts of these tissue samples were fixed in 10% formalin and the remained tissues were immediately frozen in liquid nitrogen. The histopathological investigation was performed with the remaining liver sample based on the criteria described by Frith and Ward [12] and the detailed information related to the histopathological alterations were presented in the Supplementary Information. The pathological diagnosis confirmed tissues were selected for the following experiments.

### Experimental design and statistical rationale

The precancerous tissues (P) and hepatocellular carcinoma (T) of Hras12V transgenic male and female mice (Ras-Tg) and normal liver (W) of wild-type male and female mice (Non-Tg) (9 months old males and 15 months old females, *n* = 9 for each group, total of 54 tissue samples) were used for proteomic analysis. The protein samples were individually prepared and three composite samples of each group were generated by randomly and equivalently mixing every three protein samples. Then, all pooled samples were lysed, trypsin digested, and labeled with different TMT tags, separated by HPLC, and then identified and quantified by LC-MS/MS (Additional file 1, Figure S1). The DEPs were identified (quantified in all examined samples; ratio ≥ 1.5 or ≤ 0.67, *p* < 0.05) and Western blot analysis was used for verifying the authenticity and reproducibility of the quantitative proteomics analysis. DAVID bioinformatics platform (http://david.abcc.ncifcrf.gov/) was explored for further GO and KEGG analysis for the identified DEPs.

Please see Supplementary Information for more detailed information of this section.

## Results

### Experimental design and quality test of TMT-based quantitative proteomics data

*Hras12V* transgenic hepatic tumor model mice have been identified and described in detail previously [4, 11]. Male Ras-Tg developed HCC at the age of 8 to 9 months old with a high incidence (almost 90%) and died due to the massively developed hepatic tumors at 12–14 months of age. By contrast, female Ras-Tg developed HCC over 15 months old with a low incidence (about 30%). Therefore, 9 months old males and 15 months old females of Ras-Tg carrying HCC at the same stage were selected to achieve our research objectives for identification of the underlying mechanisms involved in hepatocarcinogenesis and male bias. We performed multiplexed isobaric TMT labeling combined with LC-MS/MS approaches to quantify the proteome of tissues obtained from normal liver of wild-type (W) mice, hepatocellular carcinoma (T) and their paired adjacent precancerous (P) of Hras12V transgenic (Ras-Tg) mice in 9 months old male mice (9-M) and 15 months old female mice (15-F). That is, six groups were investigated: W of wild-type 9-M (MW) and 15-F (FW), P of transgenic 9-M (MP) and 15-F (FP), and T of transgenic 9-M (MT) and 15-F (FT). Nine individual samples were selected from each group to extract protein.

Mixing samples of experimental animal tissues in the same group is one of the most frequently used methods of the quantitative proteomics, especially for the inbred strains which are nearly identical to each other in the genomic background. In addition, in the case of Ras-Tg, the hepatic tumors were protruded from the precancerous tissues and had a clear boundary between them. It is easy to accurately sample and has a slim chance to mix the hepatic tumor tissues with the precancerous tissues. Therefore, in each group, three pooled samples were generated by random and equivalent mixing every three samples. This design ensures both biological replication and sample coverage, which authentically and reproducibly reflects the proteomic changes. Then, all pooled samples were lysed, trypsin digested, and labeled with different TMT tags, separated by HPLC, and then analyzed by LC-MS/MS. The workflow is depicted in Additional file 1, Figure S1.

**Fig. 1:**
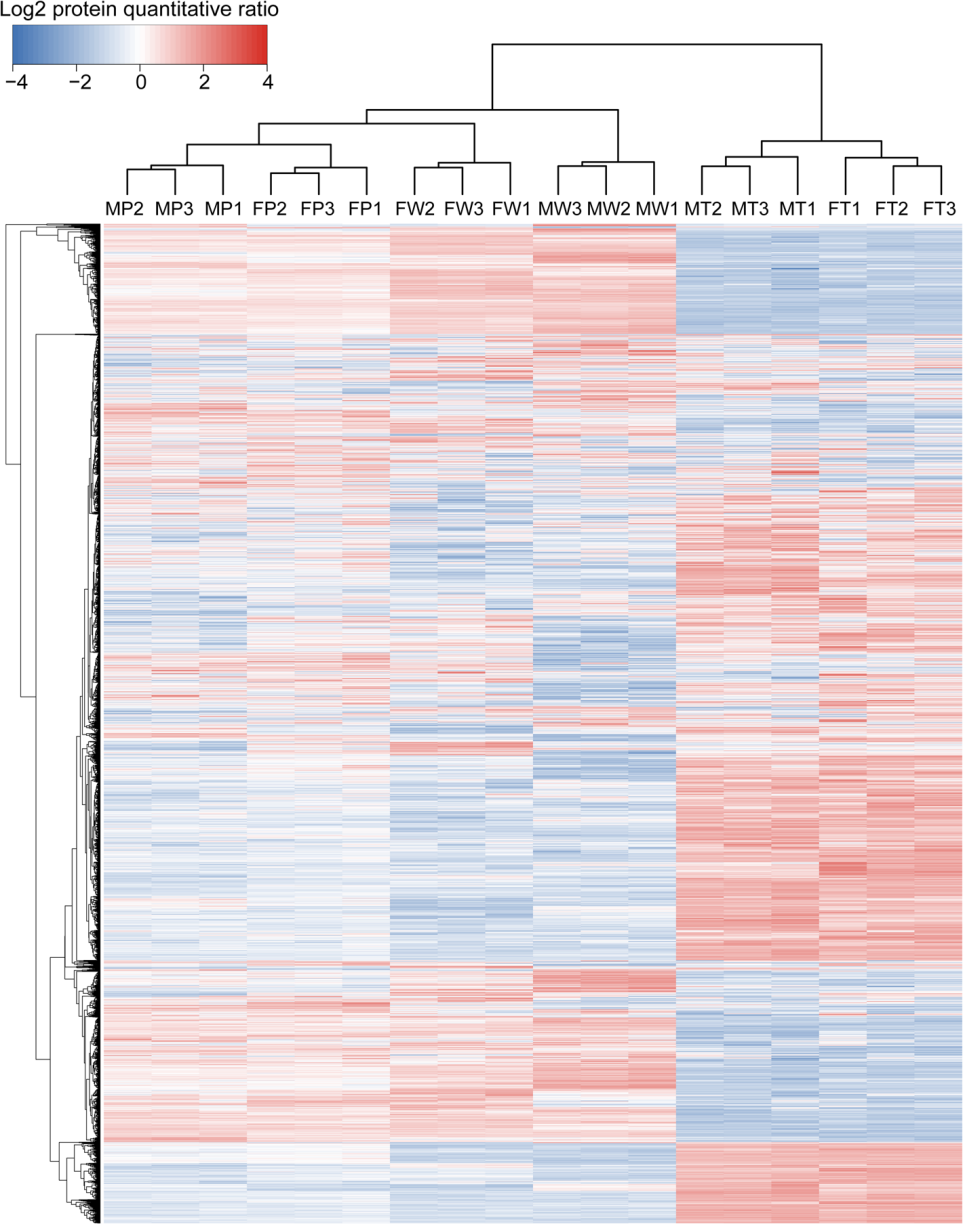
Analysis and validation of the quality of quantitative proteomics data. Unsupervised hierarchical clustering of all proteome datasets shows separation among the different groups. Protein expression levels are color-coded, showing higher and lower expression in red and blue, respectively. MW, normal liver tissues of non-transgenic 9-months-old males; MP, precancerous tissues of transgenic 9-months-old males; MT, hepatocellular carcinoma tissues of transgenic 9-months-old males; FW, normal liver tissues of non-transgenic 15-months-old females; FP, precancerous tissues of transgenic 15-months-old females; FT, hepatocellular carcinoma tissues of transgenic 15-months-old females. The numbers indicate different individuals or different composite samples

The depth of proteome coverage and quantitation is summarized in Additional file 1, Figure S2. The relative quantitative proteomes demonstrated the process of hepatocarcinogenesis by hierarchical clustering, identifying common trends in the same groups and different trends in different groups (Fig. 1). Evaluation of the reproducibility between sample pools in the same group by linear regression highlighted the strong correlation among the three independent biological replicates (Additional file 1, Figure S3).

### Identification of DEPs for hepatocarcinogenesis in males and females

**Fig. 2:**
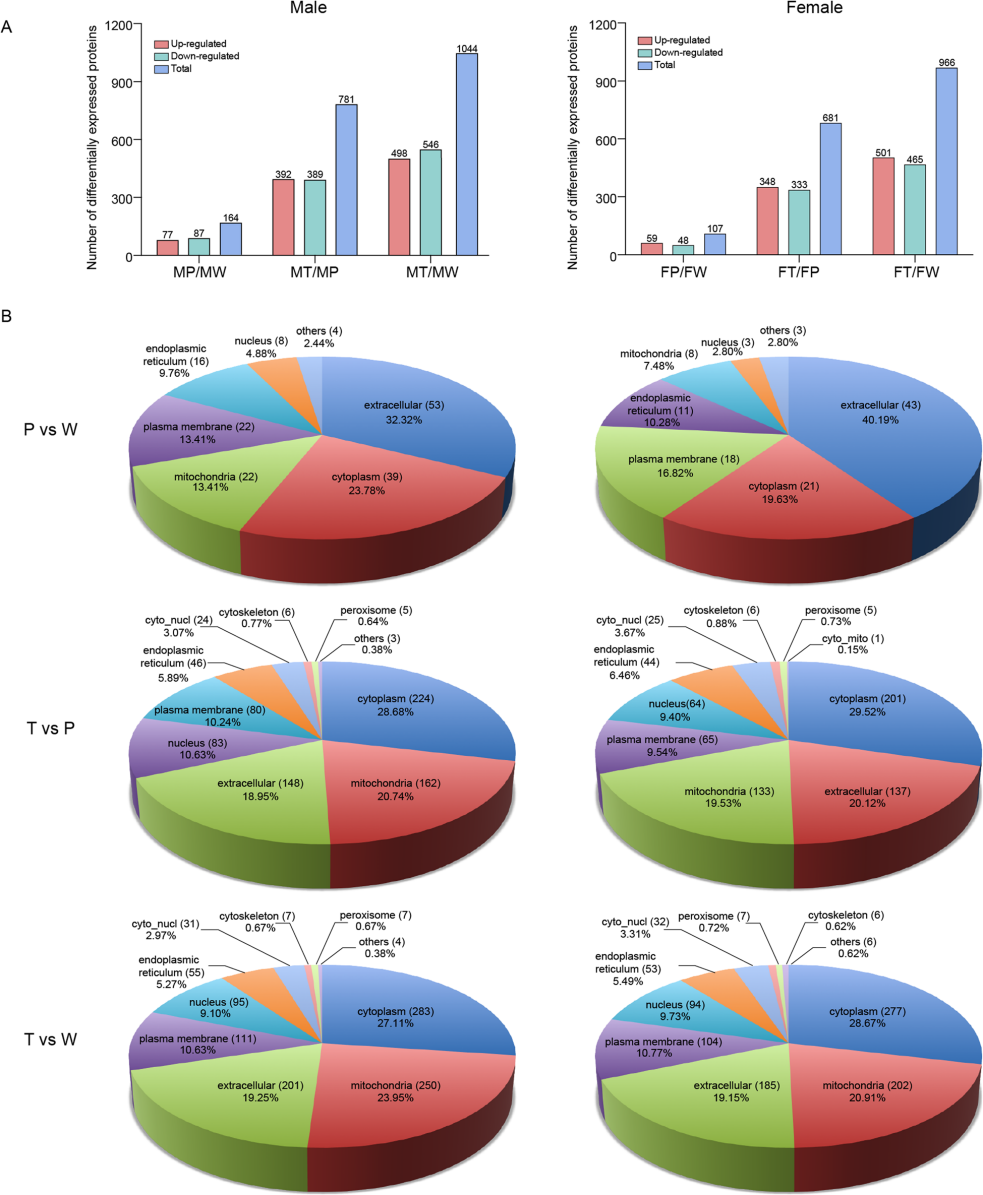
Identification of the DEPs by LC-MS/MS and subcellular location analysis. **a** Number of DEPs in paired comparisons. **b** Subcellular location analysis. The paired comparison results in males and females are shown in the left and right panels, respectively. W, normal liver tissues of wild-type non-transgenic mice; T and P, HCC tissues and their paired adjacent precancerous tissues of *Hras12V* transgenic mice. Detailed information of DEPs among W, P, and T is shown in Additional file 5, Table S4. The slash symbol means “versus”. The abbreviation and symbol definitions as well as descriptions are the same as those in the caption for Fig. 1

Totally, 5733 proteins were identified, and of which, 5193 were quantified (Additional file 2, Table S1). Finally, 1344 proteins were quantified in all examined samples, and |ratios|≥ 1.5, *p* < 0.05 were considered to be differentially expressed. A comparison of the number of the DEPs for matched pairs of W, P, and T in male and female mice is shown in Fig. 2a. Interestingly, whether comparing P versus W (P/W), T versus P (T/P), or T versus W (T/W), the number of DEPs was always higher in males than in females, which is consistent with our previous investigation [4]. These results indicate that protein expression was more easily perturbed in males than in females by oncogene activation during hepatocarcinogenesis, which reflects the significant male prevalence of HCC. Additionally, much higher numbers of DEPs in the comparisons of T/P and T/W compared with those in P/W indicate extreme changes in the hepatoma cells.

The identified DEPs were classified depending on their subcellular locations by exploring the Gene Ontology Consortium (http://www.geneontology.org/docs/go-consortium/) (Fig. 2b). The identified DEPs were found to be mainly located in the cytoplasm, mitochondria, extracellular, plasma membrane, nucleus, and endoplasmic reticulum. Notably, the proportion of subcellular located DEPs is obviously different between T/P (W) and P/W in both males and females, which indicates the particular changes in T. In particular, the significantly increased proportion of cyto_nucl and nucleus located DEPs in T reflects its important role in hepatocarcinogenesis. The obvious difference between males and females in P versus W indicates the gender-dependent responses to the Ras oncoprotein in hepatocytes. However, the largely similar distribution of DEPs in T/P (W) between sexes indicates similar pathways towards T and similar characteristics of T in males and females.

### Validation of different expression proteins by Western blotting

**Fig. 3:**
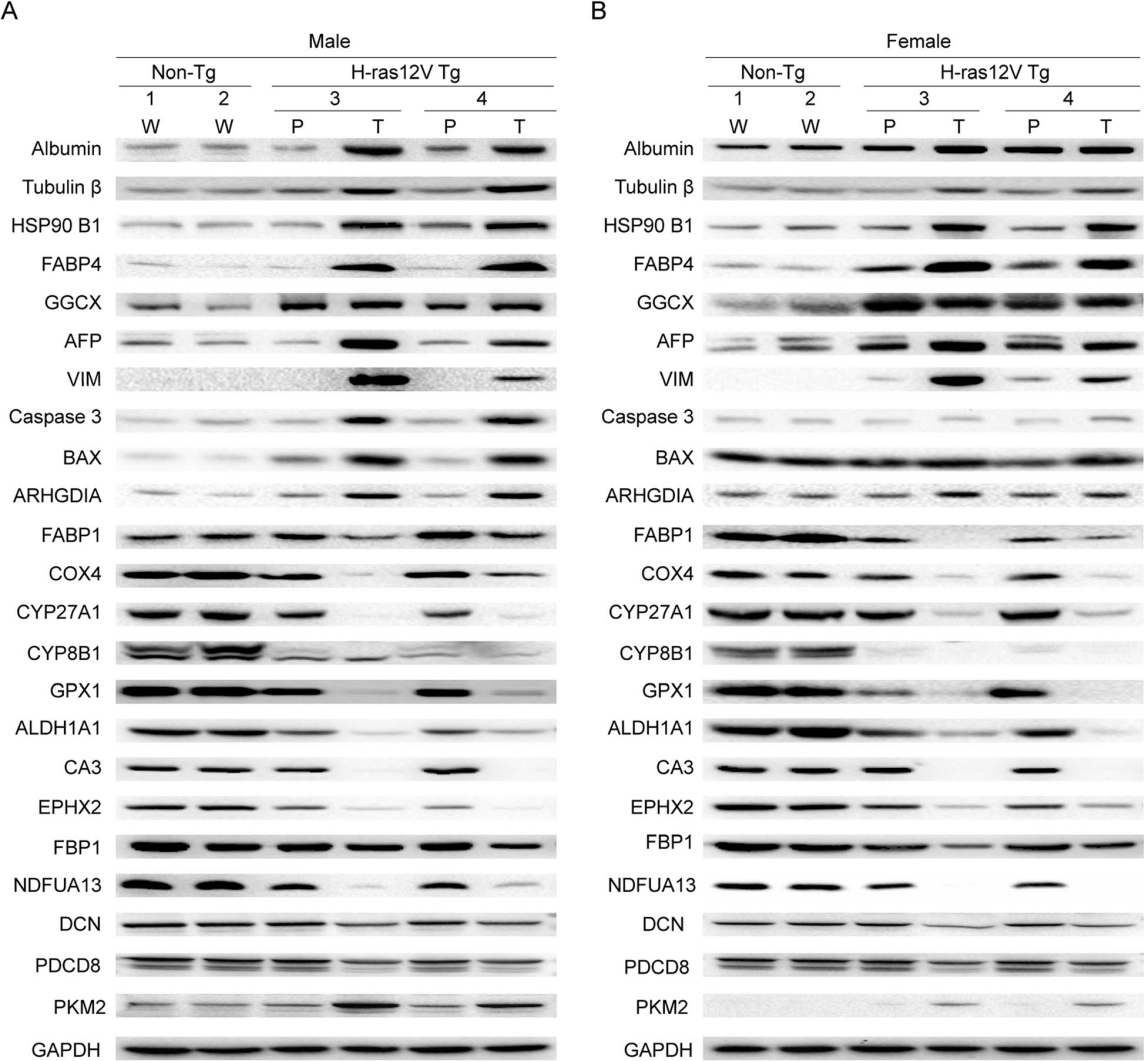
Validation of the DEPs identified by LC-MS/MS. The randomly selected twenty-three DEPs were validated in another set of samples from male (**a**) and female (**b**) of Non-Tg and Ras-Tg mice by Western blot. Non-Tg, wild type non-transgenic mice; Tg, transgenic mice. The different numbers represent different individuals. The level of GAPDH was used as a quantitative control. The abbreviation and symbol definitions as well as descriptions are the same as those in the caption for Fig. 1

To further validate the quantitative proteomics data, twenty-three of the identified DEPs with common variant trends in both sexes were further evaluated by Western blotting (Fig. 3, Additional file 1, Figure S4). The changes in these proteins expression were the same as the obtained quantitative proteomics results (Additional file 3, Table S2). The western blotting results confirmed that the proteomics analysis is convincing. Interestingly, among these randomly evaluated proteins, the expression levels of TGF-beta signaling pathway-related protein DCN, apoptosis-related proteins PDCD8, BAX, Caspase 3, and glycolysis-related protein PKM were significantly changed. It indicates that the modulations of these key cellular pathways play important roles in hepatocarcinogenesis.

### Gene ontology annotation for the hepatocarcinogenesis processes in males and females

**Fig. 4:**
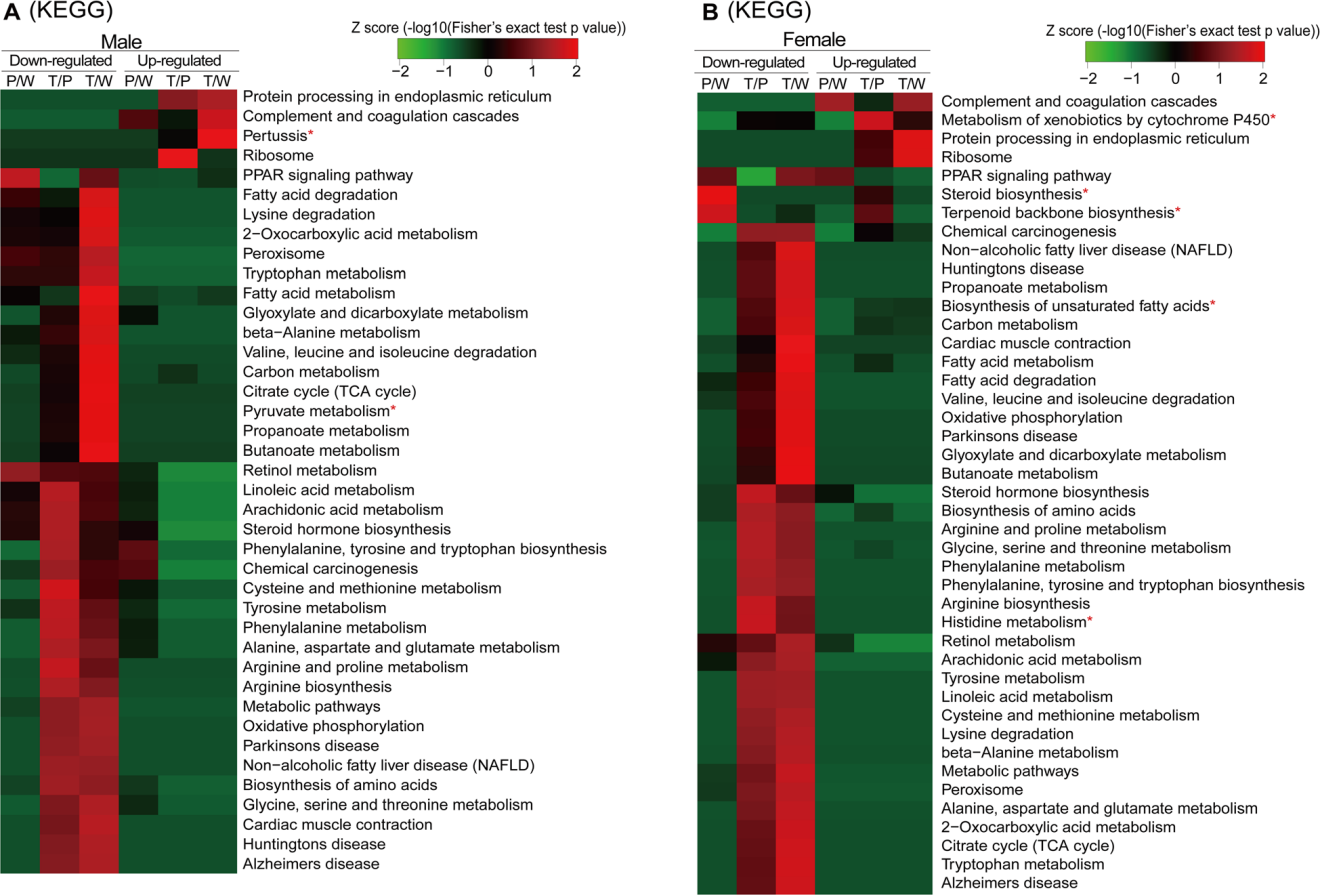
Bioinformatics analysis of DEPs during hepatocarcinogenesis in males and females. Heat map obtained from KEGG pathway analysis using DAVID bioinformatics. **a** For males and **b** for females. The slash symbol means “versus”. The abbreviation and symbol definitions as well as descriptions are the same as those in the caption for Fig. 1

To obtain more insights into the biological significance of the DEPs, the cluster enrichment analysis of Gene ontology (GO: cellular component (CC), biological process (BP), molecular function (MF)) and KEGG pathways were performed using the DAVID bioinformatics platform (http://david.abcc.ncifcrf.gov/). The detailed bioinformatics analysis results are shown in Fig. 4, Additional file 1, Figure S5 and Additional file 4, Table S3. The significantly enriched items in the up- and down-regulated DEPs show a close association with the biological processes of hepatocarcinogenesis and/or to the responses to Ras oncoprotein. The majority of common cluster-enriched items have the same variant trends between sexes, although the different enrichment strengths and different involved DEPs between sexes were shown, which indicate the common pathways towards hepatocarcinogenesis induced by the *ras* oncogene in both sexes. In particular, several commonly enriched cluster items have obviously different variant trends between males and females. For example, retinol metabolism found in down-regulated DEPs in KEGG analysis was the strongest in the P/W comparison in males, whereas it was strongest in the T/W comparison in females. The depletion of retinoid resulting from attenuated retinol metabolism pathways was common in HCC, and epidemiological and experimental reports show that the application of retinoid ATRA can prevent liver cancer [13]. These differences indicate that attenuated retinol metabolism occurs earlier in males than in females in response to *ras* oncogene expression, which may link to the male-biased hepatocarcinogenesis.

Moreover, some items are uniquely correlated to males or females, which indicate gender-dependent biological changes (marked with red asterisks in Fig. 4 and Additional file 1, Figure S5) during hepatocarcinogenesis and/or in the response to *ras* oncogene expression. Therefore, these data offer important information for further mechanism investigation of gender-dependent hepatocarcinogenesis. For example, steroid biosynthesis in KEGG cluster analysis and the steroid biosynthetic process in BP cluster analysis were uniquely and significantly enriched in the P/W comparison in down-regulated DEPs of females. Steroid biosynthesis is crucial for producing multiple important metabolites such as vitamin D, cholesterol, and steroid hormones. Steroid biosynthesis has been found to be important for hormonal dependence of cancer types, and the enzyme inhibitors for steroid biosynthesis have taken center stage as first-line therapies for breast and prostate cancer [14]. Hepatocarcinogenesis is also well-known to have a male prevalence, and the unique inhibition of steroid biosynthesis in female hepatocytes in response to *ras* oncogene expression may contribute to this sex bias.

The peroxisome proliferator-activated receptors (PPARs) form heterodimers with retinoid X receptors (RXRs), belonging to the superfamily of nuclear lipid-sensing receptors (NR) and playing a central role in modulating the expression of a set of genes involved in multiple pathways including lipid metabolism. Intriguingly, PPAR signaling pathway was the only one that was significantly cluster-enriched in both up-regulated and down-regulated DEPs only in the P/W comparison in females (Fig. 4). Further investigations showed that the up-regulated DEPs were focused on the lipogenesis and lipid transport. However, the down-regulated DEPs were focused on the cholesterol metabolism, fatty acid oxidation, and ketogenesis (Additional file 4, Table S3). The suppression of cholesterol metabolism, fatty acid oxidation, ketogenesis, and the elevation of lipogenesis would result in the accumulation of cholesterol and fatty acid in the hepatocytes. Simultaneously, the elevated lipid transport may maintain the lipid homeostasis in the hepatocytes to avoid lipotoxicity, a recognized risk factor for liver carcinogenesis [15]. It indicates that the female hepatocytes are easier to regulate the lipid homeostasis when facing the Ras oncoprotein-induced lipid metabolism disorders. Especially, FABP4 (aP2), a suggested biomarker for non-alcoholic fatty liver disease (NAFLD) and HCC [16], was up-regulated in PPAR signaling pathway by a positive feedback regulation and continually acted on PPAR signaling pathway in both sexes, which may play important roles in steatosis and carcinogenesis.

### Common and unique DEPs involved in hepatocarcinogenesis between males and females

**Fig. 5:**
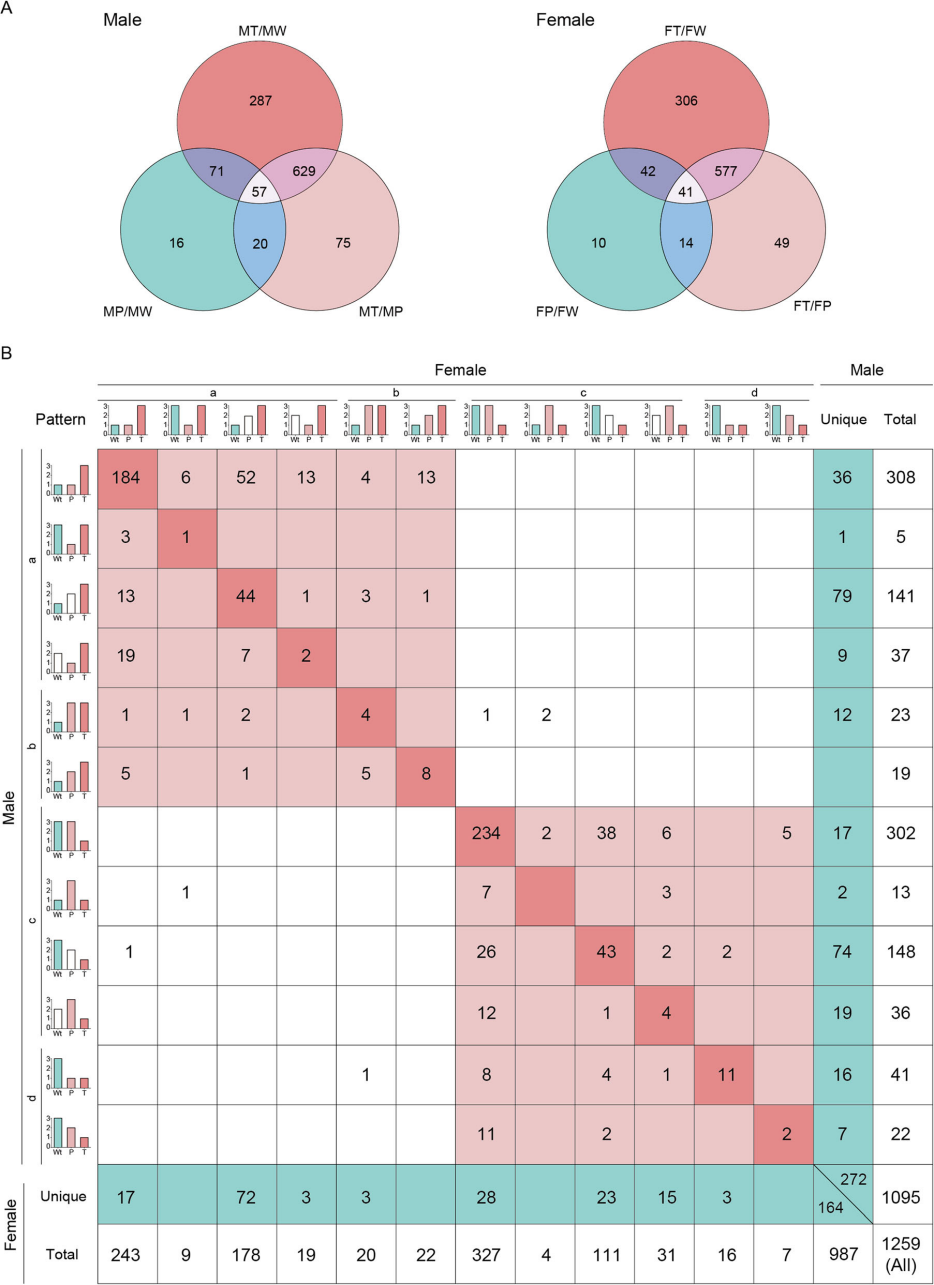
Common and unique DEPs detected during hepatocarcinogenesis in males and females. **a** Venn analysis for DEPs detected in the proteomics for males and females, respectively. The slash symbol means “versus”. **b** DEPs were divided into the different expression patterns in males and females, respectively, and then overlap the DEPs within the same pattern between males and females to determine common and unique DEPs correlated with hepatocarcinogenesis and/or the expression of the *Hras12V* oncogene. HCC positive-correlated (**a**) or negative-correlated (**c**) proteins represent that these proteins were significantly increased or decreased in T comparing with those in P and/or W, respectively. The *Hras12V* oncogene positive-correlated (b) or negative-correlated (d) proteins represent that these proteins were significantly increased or decreased in P and T when compared with those in W, respectively. In the schematic diagram of the expression pattern, the numbers 1, 2, and 3 represent the graded and significant expression levels. The colored columns represent the significantly changed expression levels comparing with at least one group in the same pattern, and the white column indicates no significant expression changes comparing with those of the other two groups. The deep red-colored boxes indicate completely overlapping DEPs between sexes in the same expression pattern. The light red boxes indicate DEPs with similar expression patterns. The blue boxes indicate unique DEPs between sexes. The abbreviation and symbol definitions as well as descriptions are the same as those in the caption for Fig. 1

To identify the common and unique DEPs involved in hepatocarcinogenesis between sexes, Venn analysis was first performed among T/P, T/W, and P/W in males and females (Fig. 5a; Additional file 5, Table S4). Then, depending on the Venn analysis, we classified the DEPs into four categories describing particular variation trends of DEPs during hepatocarcinogenesis: (a) DEPs that are positively correlated with HCC; (b) DEPs that are positively correlated with *ras* oncogene; (c) DEPs that are negatively correlated with HCC; and (d) DEPs that are negatively correlated with *ras* oncogene (Fig. 5b). Further, common and unique DEPs were identified depending on the categories (Fig. 5b; Additional file 6, Table S5).

In subsequent paragraphs, we used the “>”, “<” and “=” symbols to represent the relationships between the former and the latter tissues at protein expression levels.

Category (a): These DEPs could be further classified into four types: (1) T>P=W, (2) T=W>P, (3) T>W (P=T(W)), and (4) T>P (W=T(P)). The most proteins were classified into types (1) and (3), indicating their nonexistent or weak responses to *ras* oncogene expression in normal hepatocytes and predominantly positive roles in HCC. The DEPs classified into types (2) and (4) indicate their negative responses to *ras* oncogene in hepatocytes and may belong to the cancer defense system.

Category (b): These DEPs could be further classified into two types: (1) W<P=T and (2) W<P<T. It indicates that these DEPs are positively response to *ras* oncogene in both hepatocytes and hepatoma cells as they were increased in both P and T comparing with W.

Category (c): These DEPs could be further classified into four types: (1) T<P=W, (2) T=W<P, (3) T<W (P=T(W)), and (4) T<P (W=T(P)). The most proteins were classified into types (1) and (3), indicating their nonexistent or weak responses to oncogene expression in normal hepatocytes and predominantly negative roles in HCC. The DEPs classified into types (2) and (4) indicate their positive responses to *ras* oncogene in hepatocytes and may belong to the cancer defense system.

Category (d). These DEPs could be further classified into two types: (1) W>T=P and (2) W>P>T. It indicates that these DEPs are negatively response to *ras* oncogene in both hepatocytes and hepatoma cells as they were decreased in both P and T comparing with W.

In total, 1259 DEPs were detected. Among them, 1095 DEPs belong to males and 987 belong to females. There were 823 DEPs common to both sexes, and 272 and 164 DEPs were unique in males and females, respectively. Among the common DEPs, 393 DEPs (243 DEPS have completely the same variant trends (diagonal line)) were positively correlated to HCC, and 424 DEPs (294 DEPS have completely the same variant trends (diagonal line)) were negatively correlated to HCC. Among the unique DEPs, 137 and 95 DEPs were positively correlated to HCC in males and females, respectively, and 135 and 69 DEPs were negatively correlated to HCC in males and females, respectively.

In particular, 184 DEPs in (a)-(1) type and 234 DEPs in (c)-(1) type occupy the most proportions of DEPs in variant trend types, which represent the common DEPs especially related to HCC. The verified tumor promotion genes included in (a)-(1) type such as PKM, Tpd52, Tpm4, Ly6d, Hk2, Nqo1, Phgdh, Rab3d, Alcam, Aldh3a1, Asns, Hspa5, Hsp90b1, Txndc5, and Prkar2b and the tumor suppressor genes included in(c)-(1) type such as Rgn, Maob, Sirt3, Pbld, Pck1, Lhpp, Gnmt, Gls2, Hadh, Decr1, Hmgcs2, Fbp1, Dmgdh, Hint2, and Sdhb further confirmed the authenticity of classification analysis of the proteomic data from Ras-Tg (Additional file 6, Table S5). The remaining unverified DEPs in pools of (a)-(1) and (c)-(1) offer crucial clues for further studies to determine the mechanisms of hepatocarcinogenesis. Further bioinformatics analysis showed that pathways such as protein processing in the endoplasmic reticulum, glutathione metabolism, chemical carcinogenesis, and glycolysis/gluconeogenesis, etc. were enhanced and that pathways such as oxidative phosphorylation, citrate cycle, primary bile acid biosynthesis, retinol metabolism, steroid hormone biosynthesis, etc. were attenuated in HCC (Additional file 7, Table S6).

Additionally, 12 (in b) and 13 (in d) common DEPs in both sexes were found to be positively and negatively related to *ras* oncogene expression, respectively. Among these genes, Fn1 and SCD1 have been reported to be positively and CYP8b1 negatively related to the activation of the Ras/MAPK pathway. The remaining DEPs in these categories may be directly or indirectly regulated by Ras signaling and provide important information for further investigation.

Moreover, the number of unique DEPs (272) in males was much higher than that in females (164). Further bioinformatics analysis showed that the unique clustered KEGG pathways (*p* < 0.05) related to males including citrate cycle, oxidative phosphorylation, non-alcoholic fatty liver disease, Parkinson’s disease, lysosome, pyruvate metabolism, Alzheimer’s disease, 2-oxocarboxylic acid metabolism, and related to females including seleno compound metabolism, biosynthesis of amino acids, and chemical carcinogenesis (Additional file 8, Table S7). These analyses revealed DEPs in unique disturbed pathways under *ras* oncogene-induced hepatocarcinogenesis between sexes.

### Convergent trend during hepatocarcinogenesis between males and females

**Fig. 6:**
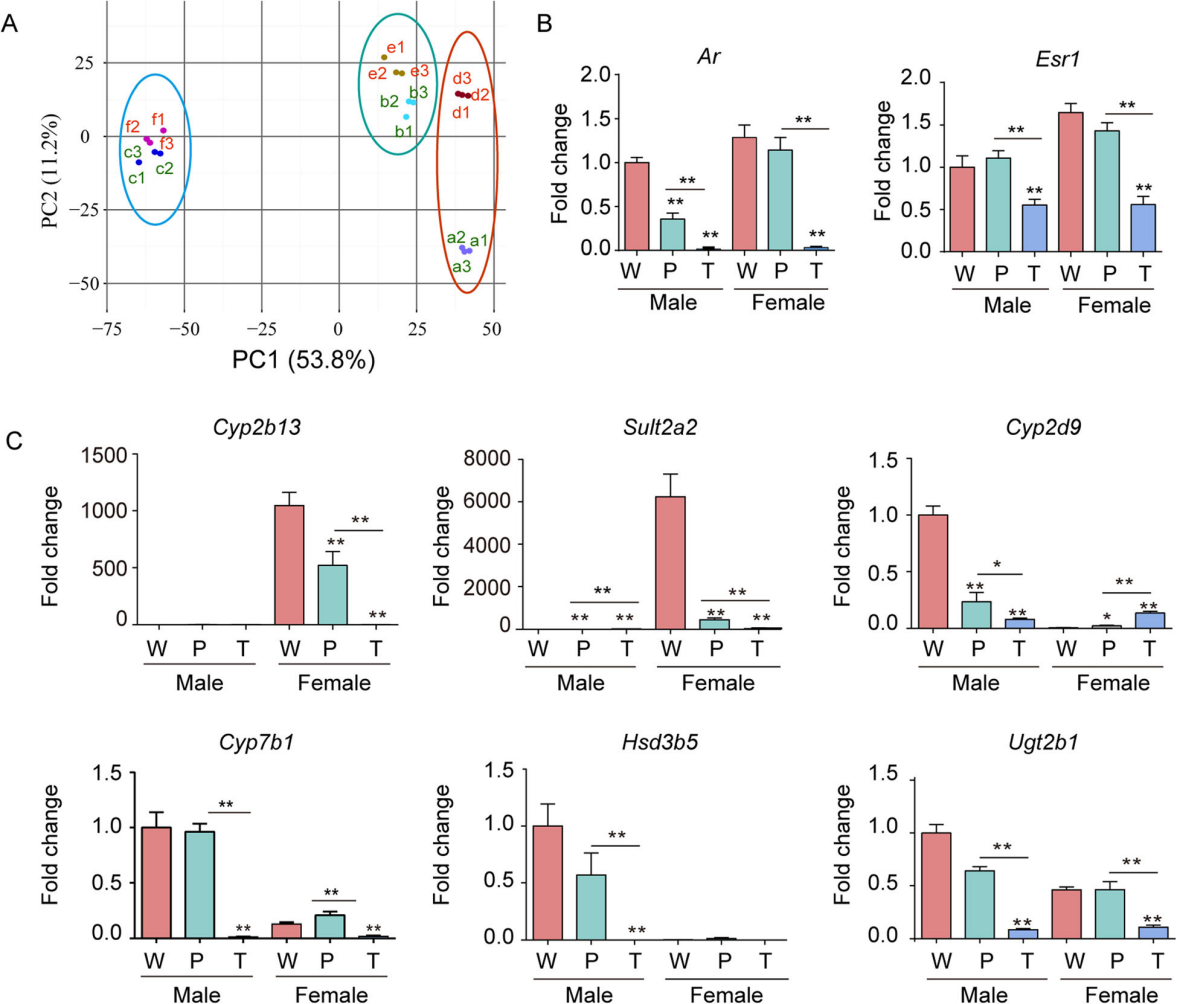
Converging trend during hepatocarcinogenesis between males and females of *Hras12V* transgenic mice. **a** Principal-component analysis (PCA) of all quantified proteins in samples (a, MW; b, MP; c, MT; d, FW; e, MP; f, MT). **b** mRNA levels of *Ar* and *Esr1* in W, P, and T of males and females as detected by RT-qPCR. **c** The mRNA levels of female-prevalent enzymes CYP2B13 and SULT2A2 and male-prevalent enzymes CYP2D9, CYP7B1, UGT2B1, and HSD3B5 in W, P, and T of male and female mice as detected by RT-qPCR. The mRNA levels of the genes were normalized to *Rp135a*. The data are expressed as the mean ± SEM (*n* = 5–7).*p < 0.05; **p < 0.01.

Principal component analysis (PCA) of all quantified proteins revealed that the Euclidean distances of sample dots between MP and MF and between MT and FT gradually shrunk and converged (Fig. 6a). In fact, this trend was also clearly demonstrated in hierarchical clustering analysis (Fig. 1) and subcellular location assays (Fig. 2b). These data indicate that the activation of Ras/ERK narrows the differences between hepatocytes of the different sexes and showed a clear converging trend in HCC between the sexes.

As the sex hormone response system has been recognized to be an important factor contributing to the difference between hepatocytes in males and females [17] and is supposed to play a crucial role in gender differences in hepatocarcinogenesis [18], we hypothesized that the sex hormone response system may be significantly altered when Ras/ERK is activated in hepatocytes and hepatoma cells. Interestingly, the mRNA levels of androgen receptor (*Ar*) and estrogen receptor 1 (*Esr1*) were significantly reduced in T of both males and females comparing with those of W and P and in P of males compared to W (Fig. 6b). Further, the different expression levels of female-biased enzymes CYP2B13, SULT2A2 and male-biased enzymes CYP2D9, CYP7B1, UGT2B1, and HSD3B5 in W were gradually eliminated in P and T between males and females based on RT-qPCR analysis (Fig. 6c). Among these genes, CYP2D9, CYP7B1, and HSD3B5 were also detected and showed the same trends in the present proteomic data (Additional file 1, Figure S6). This indicates that the *ras* oncogene plays crucial roles in regulating the responses to sex hormones in hepatocytes and hepatoma cells and hepatoma cells loss their responses to sex hormones. Anti-ERK treatment elevated the mRNA levels of *Cyp2b13* and *Sult2a2* in P of female Ras-Tg but further decreased the mRNA levels of *Cyp2d9* in P and T of both sexes of Ras-Tg (Additional file 1, Figure S7). This indicates that ERK only has a partial role in regulating the response to sex hormones and that additional downstream pathways of the *ras* oncogene are involved.

### Gender disparity in retinol metabolism during hepatocarcinogenesis

**Fig. 7:**
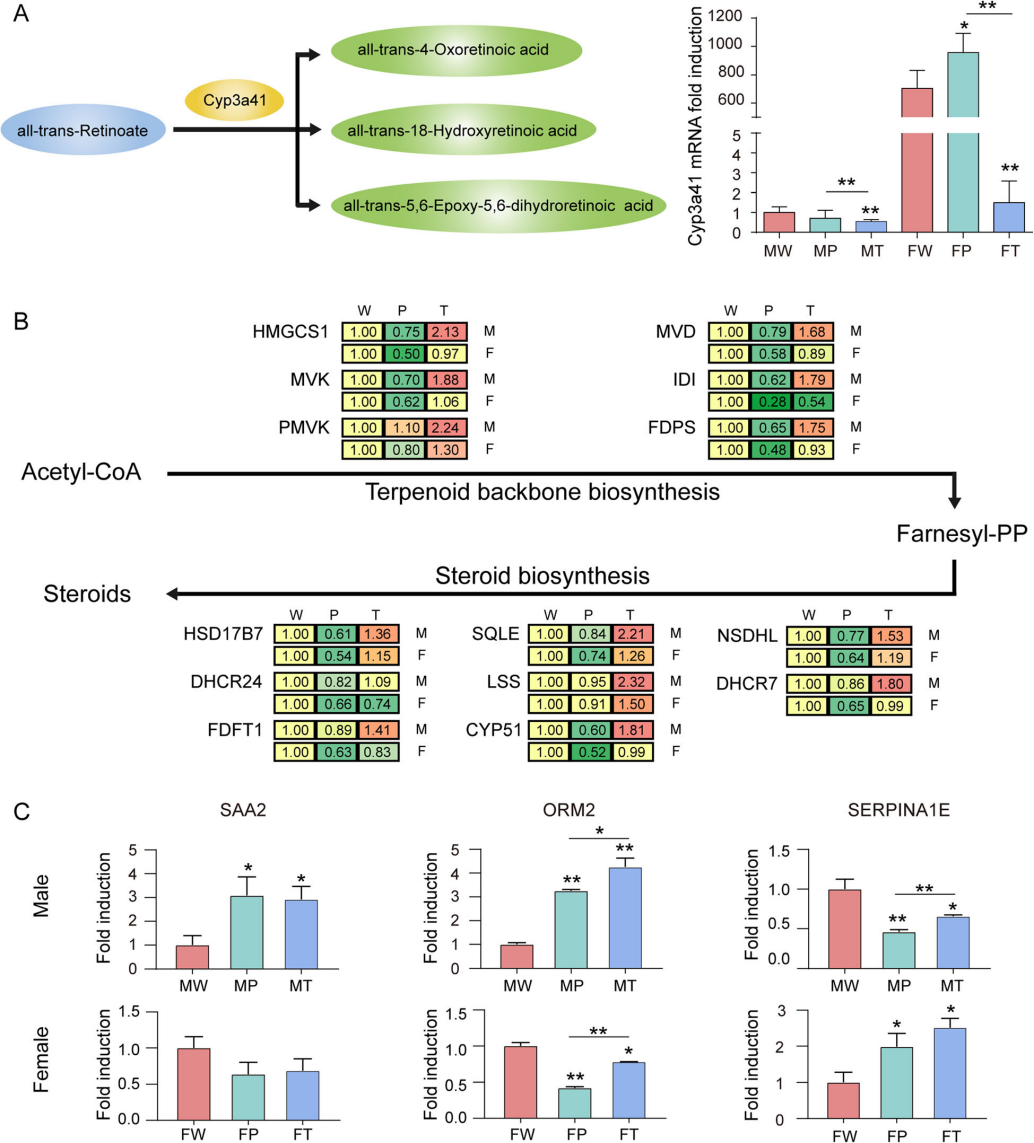
Gender disparity in metabolic pathways and biomarkers during hepatocarcinogenesis in *Hras12V* transgenic mice. **a** The mRNA levels of *Cyp3a41* in W, P, and T of males and females as detected by RT-qPCR. The mRNA levels of the genes were normalized to *Rp135a*. The data are expressed as the mean ± SEM (*n* = 5–7). **b** The expression levels of key enzymes in terpenoid backbone biosynthesis and steroid biosynthesis pathways identified by TMT-based quantitative proteomic analysis. **c** The expression levels of SAA2, ORM2, and SERPINA1E identified by TMT-based quantitative proteomic analysis. *p < 0.05; **p < 0.01. The abbreviation and symbol definitions, as well as descriptions, are the same as those in the caption for Fig. 1

Retinol metabolism is significantly inhibited in HCC and multiple metabolites derived from it have shown potential anti-cancer effects [13]. Interestingly, we found that the expression patterns of important enzyme CYP3A41 in retinol metabolism were significantly different in W and P between males and females. Proteomics data showed that CYP3A41 was abundantly expressed in the hepatocytes of females but hardly detected in that of males. When facing the expression of Ras oncogene, the expression of CYP3A41 was further elevated in females but not showed significantly changed in males (Fig. 7a). These results were further confirmed at mRNA levels. However, CYP3A41 was hardly detected in HCC tissues of both females and males (Fig. 7a). It indicates that CYP3A41 may play important roles against hepatocarcinogenesis. Abundant expression of CYP3A41 and its further elevation when facing Ras oncogene may play important roles in protecting females from hepatocarcinogenesis.

### Gender disparity in terpenoid backbone biosynthesis and steroid biosynthesis during hepatocarcinogenesis

Terpenoid backbone biosynthesis and steroid biosynthesis are basic pathways for synthesis of multiple bioactivators and their significant changes had been frequently detected in multiple cancer types including HCC. Intriguingly, our proteomics data showed that these two pathways were significantly and differently influenced between males and females during hepatocarcinogenesis. Terpenoid backbone biosynthesis and steroid biosynthesis-related multiple key enzymes were significantly down-regulated in P compared with W in females (fold change <0.67; *P* < 0.05). However, these are less likely to occur in males (Fig. 7b). It indicates that the two pathways are more inhibited in females than in males when facing Ras oncogene expression in hepatocytes. Interestingly, most of these enzymes were up-regulated in T compared with W in males (fold change >1.5; *P* < 0.05). However, these were rarely happened in females, although most of these enzymes restored their expression levels compared to W (Fig. 7b). It indicates that the up-regulation of the activities of the two pathways is essential for hepatocarcinogenesis. Therefore, the different expression changes of these key enzymes between sexes may contribute to the gender disparity in hepatocarcinogenesis.

### Gender disparity in biomarkers for Ras oncogene expression and HCC

Gender-dependent biomarkers for HCC and oncogene expression are not well established. By exploring the database in the present study, we found that SAA2, ORM2, and SERPINA1E had different expression patterns in Ras oncogene expressing hepatocytes and HCC of males and females. For SAA2 and ORM2, they were significantly up-regulated in P and T compared with W in males, but significantly or obviously down-regulated in P and T compared with W in females (Fig. 7c). For SERPINA1E, it was significantly down-regulated in P and T compared with W in males, but significantly up-regulated in P and T compared with W in females (Fig. 7c). These results indicated that these proteins are differently responded to Ras oncogene expression and hepatocarcinogenesis in different gender background, therefore may be potential gender-dependent biomarkers for detecting HCC and activated oncogene.

## Discussion

Although the gender disparity and the important roles of Ras/MAPK signaling pathway in hepatocarcinogenesis have been well recognized, the involved global biological changes and underlying molecular mechanisms remain to be elucidated. Omics are powerful techniques providing an integral and systemic view of both physiological and pathological processes and give potential and functional insights for further investigation. However, because of the complex pathogenesis and symptoms that result in difficulty in collecting matched specimens, systemic investigations based on gender disparity and Ras/MAPK activation using omics have not been reported in clinical HCC. *Hras12V* transgenic mice with conforming characteristics such as the same genetic background and oncogenic etiology, gender disparity in the hepatocarcinogenesis incidence and development, offer a suitable animal model for dissecting the underlying mechanisms. By using proteomics, the systemic detection of common and unique DEPs during hepatocarcinogenesis between sexes and further verification and bioinformatics analysis were performed by exploring *Hras12V* transgenic mice, which revealed multiple interesting points.

The liver is a sexually dimorphic organ with gender disparity in gene expression, mitochondrial function, microsomal enzyme activity, membrane lipid composition, immune response, and metabolism of drugs and toxins [19, 20]. The present data confirm the sexually dimorphic liver in protein expression profiles between wild-type males and females (Fig. 6). A HCC induced by the *ras* oncogene initiated in sexually dimorphic situations may contribute to the gender disparity in hepatocarcinogenesis. In particular, the sex hormones and their receptor-regulated genes play crucial roles in the sexually dimorphic liver and are therefore recognized as potential factors facilitating male-biased hepatocarcinogenesis. The sex hormone response genes presented in the detected DEPs in livers between wild-type male and female mice indicate reasonable sampling and reliable data for elucidating the underlying mechanisms of gender disparity in hepatocarcinogenesis in the present experimental system.

The fact that there is no difference in HCC incidence between men and postmenopausal women indicates that estrogen protects but that androgen promotes hepatocarcinogenesis. However, despite the fact that gender has a strong impact on HCC risk, conflicting therapeutic effects were showed on the advanced HCC patients by targeting sex hormone pathways [21–24]. It has been suggested that the variant estrogen receptors occurred in clinical HCC may lead to non-response to estrogen [25]. Interestingly, in the *Hras12V*-induced HCC, the mRNA levels of androgen and estrogen receptors were significantly down-regulated in both sexes, and consistently, the sex-responsive genes were also significantly down-regulated in males and females (Fig. 6), which leads to a minimal difference in protein expression profiles between HCC tissues of males and females. This novel evidence indicates that after the onset of a HCC, the loss of sex hormone responsiveness may lead to ineffective therapeutic approaches targeting sex hormone-related pathways.

The DEP profiles of precancerous tissues surrounding HCC offer crucial information for hepatocarcinogenesis. As the Ras signaling pathway was activated in various liver diseases including hepatitis and cirrhosis, which build the fundamental process for HCC, investigation of precancerous liver tissues expressing the *ras* oncogene will provide important insights into changes in multiple biological processes inducing by the *ras* oncogene. The biological changes in precancerous liver tissues are in a complex, disordered situation. Some DEPs may couple with the *ras* oncogene to contribute to hepatocarcinogenesis whereas other DEPs may be the responses of defense systems to the expression of Ras oncoprotein. Therefore, precancerous liver tissue may represent the balance between defense system and carcinogenesis-driving forces.

In particular, the Ras pathway has been shown to influence sex hormone response in several tissues and pathological situations. In the present study, *ras* oncogene expression reduced the gender disparity in hepatocytes by attenuating sex hormone-related pathways and thereby attenuating the influence of sex hormones on hepatocarcinogenesis. Additionally, the inhibition of p-ERK only affects some sex hormone response target genes, indicating that ERK has only a partial role in influencing sex hormone pathways and that other Ras-related pathways also have a functional role.

Interestingly, although there is a significant difference in the proteomic profile in W between sexes, the differences were reduced along with the comparison in P and T between sexes (Fig. 6). In addition, although originating from different gender backgrounds, the variation tendencies in the biological process along with hepatocarcinogenesis are largely common (Additional file 1, Figure S5). This indicates that although there is the gender disparity in hepatocarcinogenesis, the common disordered pathways are essential in the process of hepatocarcinogenesis in both sexes, which offers crucial information regarding the barrier for hepatocarcinogenesis and gender disparity in overcoming this barrier. These data provide useful clues for both common and different sex clinical prevention and therapy strategies.

Gender differences and therapeutic strategies have undergone very few analyses. A report showed that the responses to anti-HCV therapy based on Peg-IFN and ribavirin administration are poorer in females than in males greater than 50 years old, indicating that older women with low estrogen levels could be responsible for the impaired response to therapy [26]. Therefore, gender disparity in drug susceptibility reflects sex-biased expression of hepatic enzymes involved in the metabolism of drugs. In the present study, the detected DEPs in normal liver, liver harboring the *ras* oncogene, and hepatoma induced by *ras* oncogene between males and females were mainly focused on metabolic pathways (Fig. 4). These metabolic differences could contribute to the gender disparity in hepatocarcinogenesis, clinical outcome, and drug efficacy and toxicity.

Retinoids, a group of structural and functional analogs of vitamin A, exert their biological function on the regulation of epithelial cell growth, differentiation, and development primarily through two distinct nuclear receptors: retinoic acid receptors (RARs) and retinoid X receptors (RXRs) [27, 28]. The depletion of retinoids and abnormalities in the expression and function of RARs and RXRs are highly associated with the development of various cancers, including HCC. In particular, the depletion of retinoic acid (RA), an active metabolite of retinoids, and a malfunction in RXRα due to phosphorylation by the Ras/MAPK signaling pathway are profoundly associated with the development of HCC and thus may be a critical target for HCC chemoprevention [27–29]. We have previously reported the depletion of RA and significantly phosphorylated RXRα in *Hras12V*-induced HCC [9]. In the present study, the proteomic analysis data again focus on significant changes in retinol metabolism, which is significantly inhibited in HCC tissues in both sexes (Fig. 4). This indicates that the disorders of retinol metabolism may be important events in hepatocarcinogenesis and development. Interestingly, retinol metabolism was also strongly inhibited in P of males but not in P of females in the paired comparison between W and P of males and females, respectively (Fig. 4), which indicates that retinol metabolism may contribute to the sex disparity in hepatocarcinogenesis. This speculation is further evidenced by the comparison between sexes in W, P, and T. Significant differences were found between males and females in W and P but not in T (data not shown). Especially, retinol metabolism-related important enzyme CYP3A41 is extremely higher expressed in females compared with males and further up-regulated when facing Ras oncogene expression (Fig. 7a). Interestingly, the all-trans-retinoic acids (ATRAs), the products of Cyp3a41, have exhibited their anti-cancer effects and been included in clinical anti-tumor therapeutical schemes [30]. This implies that the disparity of retinol metabolism between sexes in normal liver tissues and precancerous liver tissues may contribute to the disparity in hepatocarcinogenesis. Further investigation in the gender disparity of retinol metabolism and its contribution to hepatocarcinogenesis will eventually improve gender-related individualized therapy.

Steroids are complex and exert crucial physiological and pathological roles in endocrine, paracrine, and intracrine actions through binding corresponding receptors [31]. Recently, steroid metabolomics has been proved to be a novel tool for detecting diseases including cancer [32, 33]. Terpenoid backbone biosynthesis and steroid biosynthesis are essential routes for producing multiple steroids such as cholesterol, steroid hormones, vitamin D2, vitamin D3, etc. Apart from their crucial roles in breast and prostate cancer [34, 35], the key enzymes such as Pmvk, Fdps, Sqle involved in the two pathways are frequently up-regulated in multiple cancer types including HCC depending on the TCGA and GTEx database (http://gepia.cancer-pku.cn). Inhibition of steroid biosynthesis has been suggested to be a potential anti-HCC therapeutic strategy [36]. In the present study, we found that the expression patterns of key enzymes involved in the two pathways are significant gender disparity during ras oncogene-induced HCC. The higher expression of the two pathways related enzymes in males compared with those in females (Fig. 7b) may contribute to male prevalence in hepatocarcinogenesis.

Steroid hormones such as testosterone, progesterone, cortisol, aldosterone, and estradiol are important endocrine chemical messengers that are involved in a vast number of physiological processes including metabolism, inflammation, electrolyte and fluid balance, reproduction, and secondary sex differentiation [37, 38]. Steroid hormones are synthesized from cholesterol by members of the cytochrome P450 superfamily of monooxygenases and steroid dehydrogenases. Although very few tissues contribute substantially to de novo steroidogenesis, primarily the adrenal glands, gonads, and placenta, steroids are extensively metabolized, primarily in the liver by many transformations that generally reduce their activities [39]. As the liver is a hormone-sensitive organ, liver cells respond to sex hormones during treatment for inactivation and excretion. The steroid machinery is expressed in a development- and tissue-specific manner with the expression of these factors being tightly regulated by an array of transcription factors [40]. In liver diseases including HCC, the significant changes in steroid hormone biosynthesis have been recently defined [41–44], which indicates its potential roles in the pathological progress of liver diseases. In this study, steroid hormone biosynthesis was also found to be significantly changed during hepatocarcinogenesis in both sexes (Fig. 4). This indicates that disorders of steroid hormone biosynthesis may be important events in hepatocarcinogenesis. Interestingly, the comparison between sexes showed that steroid hormone biosynthesis was significantly different between sexes in W and P but not in T (Fig. 4). This implies that the gender disparity of steroid hormone biosynthesis in normal liver tissues and precancerous liver tissues may contribute to the disparity of hepatocarcinogenesis. This evidence provides further clues for investigating steroid hormone biosynthesis roles in liver diseases.

Biomarkers with gender disparity for HCC and oncogene expression have not been well established. SAA2 and ORM2 had been reported to be significantly up-regulated in HCC patients and been suggested as potential biomarkers [45–47]. For SERPINA1, the polymorphisms, fucosylation, and up-regulation of its isoforms have been suggested to be related to hepatocarcinogenesis, metastasis, and sorafenib-resistant [48–50]. However, whether these biomarkers possess gender disparity is not investigated. In the present study, we found that the expression patterns of SAA2, ORM2, and SERPINA1E are significantly different in male and female mice when facing ras oncogene expression and during hepatocarcinogenesis (Fig. 7c). These evidences provide important clues for exploring gender-dependent biomarkers for clinical HCC diagnoses.

We previously reported a 2D-DIGE analysis for hepatic tumorigenesis in Hras12V transgenic mice [4]. In that report, 89 DEPs were finally identified. Among them, 81 proteins were detected in this study; 54 proteins showed significant changes and 26 proteins have the same variant trends among W, P, and T in males and females (Additional file 9, Table S8). These results showed not only high consistency between the two studies but also obvious differences due to the different methodologies.

TMT-based multiplexed relative quantification has some advantages including a reduced overall experiment time and experimental variance, increased sample throughput, and fewer missing quantitative values among samples, less sample material required, and significantly increased coverage (tens of thousands of proteins) [51, 52]. Although having lower coverage of small, large, basic, and hydrophobic proteins, being time-consuming and difficult to automate, 2D-DIGE analysis can efficiently separate and identify the different protein isoforms such as functional cleavage, acetylation, and glycosylation that warrant further investigation [53–55]. Therefore, our previous studies employing 2D-DIGE analysis largely reflect the different expression of protein isoforms, while the present studies using TMT-based multiplexed relative quantification only reflect the changes of protein level-summed total isoforms. The two studies reveal important information from different angles. This indicates that the two proteomic techniques are complementary, resulting in the identification of common and distinct expression changes with a reasonable agreement in the direction of altered expression for the common proteins identified [55].

In addition, in our previous study, the metabolite profiles of HCC in male Ras-Tg were characterized by GC-TOF-MS analysis [56]. The results showed the significant alterations of glycolysis, pentose phosphate pathway, tricarboxylic acid cycle, lipid biosynthesis, glutathione metabolism pathway and the bile acid metabolism pathway, etc. in HCC comparing with the wild-type liver tissues. Consistent with the metabolomic data, the present proteomics data indicated that comparing with the wild-type liver tissues, in male HCC: (1) the glycolysis-related key enzymes PFK and PKM, the pentose phosphate pathway-related key enzyme G6PD, the tricarboxylic acid cycle-related key enzyme ACLY, and the lipid biosynthesis-related key enzymes FASN, LSS, DHCR7, and MVD were significantly up-regulated; (2) the glutathione metabolism pathway-related key enzyme ABAT and the bile acid biosynthesis-related key enzymes CYP8B1, CYP27A1, and BAAT were significantly down-regulated (Additional file 10, Table S9). Therefore, these data indicated that the metabolomic data informed functional interpretations of proteomic results and the proteomic data helped to a better understanding of metabolomics results by emphasizing the involvement of the key enzymes.

Moreover, compared with our transcriptomic data, which could detect over 30,000 genes and 10,000 differentially expressed genes [56], the global proteomics data have limits in the detection of lower abundance proteins. This is why the detected proteins in the present study are enriched in high abundance proteins such as enzymes, which results in the revealed changes largely focusing on metabolic pathways. In addition, the strict scale in the present study to only analyze the proteins detected in all examined samples ruled out some important DEPs such as cancer-related proteins, which changed dramatically during carcinogenesis, such as Cacna1b, Col15a1, Hmgcr, Zfp82, and Znrd1-as. Along with technical progress, and by exploring enriched and specialized proteomics such as phosphorylation, further in-depth exploration of proteomic changes during hepatocarcinogenesis and in gender disparity will provide more meaningful clues.

In this paper, the objective of our study was to analyze proteomic differences in gender-dependent hepatocarcinogenesis in Ras-Tg. Due to 6 months late for female Ras-Tg mice to develop HCC compared with male Ras-Tg mice, it was necessary to select 9-month-old males and 15-month-old females of Ras-Tg to collect the HCC tissues and their adjacent precancerous liver tissues for achieving the research objective. However, age could be also a factor to drive the proteomics differences and this defect was unavoidable and the age-related protein changes could not be detected in the present experimental system. Nevertheless, the different expression levels of male-prevalent enzymes CYP2D9, CYP7B1, UGT2B1, HSD3B5, and female-prevalent enzymes CYP2B13 and SULT2A2 in livers of 9-month-age males and 15-month-age females of wild type mice, respectively, indicate the obvious gender disparity in the physiological status (Fig. 6c). Therefore, the proteomics data and comparative analysis in the present study could basically represent the sex differences in hepatocarcinogenesis and development.

Androgen/androgen receptor (AR) signaling plays important roles in the progression of liver diseases and has been proven to promote the HCC initiation at early-stage but suppress the HCC progression at late-stage [57–60]. AR is expressed in hepatocytes of both males and females; therefore, its ligand androgen has been recognized as a risk factor for HCC [61, 62]. Consistently, our report showed that orchiectomy significantly reduced serum testosterone level and hepatic tumorigenesis of Ras-Tg males [63] and artificially increased serum testosterone levels of Ras-Tg females significantly promoted the hepatotumorigenesis (data not show). Although the overexpression or variant of AR has been considered to play a dominant role in HCC [64–66], others showed decreased expression of AR in HCC [57, 67, 68]. These conflicting results regarding the expression and role of AR in HCCs required more detailed investigations [58]. In the present study, although the AR expression was moderately reduced in precancerous tissues of Ras-Tg males compared with that of Ras-Tg females, the significant gender disparity in precancerous tissues between males and females indicates the effective role of androgen/AR signaling in Ras-Tg males for promoting HCC initiation (Fig. 6). However, the extremely reduced AR expression in HCC of both males and females of Ras-Tg may indicate its suppressive role in the HCC progression (Fig. 6). The precise underlying mechanisms remained to be further investigated.

## Conclusion

This study presents for the first time global proteomics data regarding *Hras12V*-induced hepatocarcinogenesis with gender disparity. The common, unique, and systemic signatures found in the protein expression profiles of male-biased hepatocarcinogenesis will provide important information to the database of cancer-related proteins and crucial clues for further revealing the underlying mechanisms. Especially, the common and unique DEPs detected in precancerous hepatocytes and hepatoma cells of male and female Ras-Tg offer important candidate biomarkers for detecting the activation of Ras signaling pathway and HCC in clinical diagnosis. Additionally, the convergent trend during hepatocarcinogenesis between males and females indicates the homogeneity and the loss of sex hormones responses of HCC which reflects the little effect for therapeutic strategies targeting sex hormone systems for clinical HCC patients.

### Perspectives and significance

The present study provides for the first time comprehensive proteome profiles and novel insights in male-bias hepatocarcinogenesis induced by the *Hras12V* oncogene. Comparison and bioinformatics analyses within W, P, and T of males and females showed the common and unique DEPs and cluster enriched items between sexes, which indicates the common and gender disparity pathways towards HCC. Apart from common DEPs, the number of unique DEPs (272) in males was much higher than that (164) in females. Consistently, further bioinformatics analysis showed more disturbed pathways in males than in females during *ras* oncogene-induced hepatocarcinogenesis. These findings indicate not only the gender-dependent biological processes in hepatocarcinogenesis but also the gender-dependent molecular responses to oncogenes in hepatocytes. We, therefore, hypothesized that female hepatocytes are much more difficult to be disturbed by oncogenes while male hepatocytes readily to do so. An innovative expression-change-pattern analysis further categorized the common and unique DEPs into HCC and *ras* oncogene positive/negative-related DEPs. These database revealed not only the HCC related but also the *ras* oncogene responded DEPs and pathways in hepatoma cells and/or hepatocytes of both sexes, which offer valuable insights for clinical investigation. In addition, it showed that the *ras* oncogene gradually and significantly reduced the response to sex hormones from hepatocytes to hepatoma cells in both males and females and therefore shrunk the gender disparity between males and females. This novel evidence indicates that after the onset of a HCC, the loss of sex hormone responsiveness may lead to ineffective therapeutic approaches targeting sex hormone-related pathways. The common, unique, and systemic signatures found in the protein expression profiles of male-biased hepatocarcinogenesis will provide important information to the database of cancer-related proteins and crucial clues for further revealing the underlying mechanisms. Additionally, the common and unique DEPs detected in precancerous hepatocytes and hepatoma cells of male and female Ras-Tg offer important candidate biomarkers for detecting the activation of the Ras signaling pathway and HCC in clinical diagnosis.

## Supplementary information

**Additional file 1: Figure S1.** Experimental flowchart. Three sets of biologically replicate composite samples labeled with TMT (6-plex) were separated by HPLC and analyzed by LC-MS/MS. Proteins were identified using Maxquant software. After validation, the identified differentially expression proteins (DEPs) underwent bioinformatics analysis. MW, normal liver tissues of non-transgenic 9 months old males; MP, precancrous tissues of transgenic 9 months old males; MT, hepatocellular carcinoma tissues of transgenic 9 months old males; FW, normal liver tissues of non-transgenic 15 months old females; FP, precancerous tissues of transgenic 15 months old females; FT, hepatocellular carcinoma tissues of transgenic 15 months old females. The numbers indicate different individuals or different composite samples.

**Additional file 2: Figure S2.** Depth of proteome coverage and quantitation for the three composite samples. (*A*) Mass offset distribution of peptides, (*B*) length distribution of peptides.

**Additional file 3: Figure S3.** Heatmap of the Pearson’s correlation (R2) of the proteome dataset.

**Additional file 4: Figure S4.** The presentation of images of original membrane for Western blot.

**Additional file 5: Figure S5.** Bioinformatics analysis of DEPs during hepatocarcinogenesis in males and females.

**Additional file 6: Figure S6.** Expression levels of CYP2D9, CYP7B1, and HSD3B5 identified by TMT-based quantitative proteomic analysis. W, wild-type liver tissues; P, precancerous tissues of transgenic mice; T, hepatocellular carcinoma tissues of transgenic mice. The data are expressed as the mean ± SEM (n = 3) (**, *p* < 0.01).

**Additional file 7: Figure S7.** Changes in mRNA levels of *Cyp2b13, Sult2a2, Cyp2d9, Cyp7b1, Hsd3b5,* and *Ugt2b1* in P and T treated by the ERK inhibitor AZD6244. P, precancerous tissues of transgenic mice; T, hepatocellular carcinoma tissues of transgenic mice. The mRNA levels of genes were detected by RT-qPCR and normalized to *Rp135a*. The data are expressed as the mean ± SEM (n = 5-6). (*, *p* < 0.05; **, *p* < 0.01).

**Additional file 8: Table S1.** Detailed information for identified and quantified proteins.

**Additional file 9: Table S2.** Detailed information of identified DEPs validated by Western Blot assays.

**Additional file 10: Table S3.** Bioinformatic elaboration for functional GO term and KEGG pathway enrichment via DAVID analysis.

**Additional file 11: Table S4.** Detailed information of DEPs by paired comparisons among W, P, and T in males and females.

**Additional file 12: Table S5.** Common and unique DEPs based on the categories.

**Additional file 13: Table S6.** Clustered KEGG pathway assay for the common DEPs based on the categories in males and females.

**Additional file 14: Table S7.** Clustered KEGG pathway assay for the unique DEPs based on the categories in males and females.

**Additional file 15: Table S8.** The comparison of results obtained by 2D-DIGE and TMT labeling methods.

**Additional file 16: Table S9.** The significantly regulated enzymes in the proteomic data corresponding to our previous metabolomic data.

**Additional file 17: Table S10.** Primer sequences for RT-qPCR.

**Additional file 18: Table S11.** Detailed information for the primary and secondary antibodies.

## Data Availability

The mass spectrometry proteomics data have been deposited to the ProteomeXchange Consortium (http://proteomecentral.proteomexchange.org) via the PRIDE partner repository with the dataset identifier PXD012410.

## Abbreviations

BP: Biological process
CC: Cellular component
F: Females
FW: Normal liver tissues of non-transgenic 15-months-old female mice
FP: Precancerous tissues of transgenic 15-months-old female mice
FT: Hepatocellular carcinoma tissues of transgenic 15-months-old female mice
HCC: Hepatocellular carcinoma
9-M: 9-months-old male mice
15-F: 15-months-old female mice
DEPs: Differentially expressed proteins
GO: Gene Ontology
KEGG: Kyoto Encyclopedia of Genes and Genomes
LC-MS/MS: Liquid chromatography-tandem mass spectrometry
M: Male
MF: Molecular function
MS: Mass spectrometry
MP: Precancerous tissues of transgenic 9-months-old male mice
MW: Normal liver tissues of non-transgenic 9-months-old male mice
MT: Hepatocellular carcinoma tissues of transgenic 9-months-old male mice
non-Tg: Non-transgenic mice
P: Precancerous tissues of Ras-Tg
P/W: P versus W
T: HCC of Ras-Tg
T/P: T versus P
T/W: T versus W
Ras-Tg: *Hras12V* transgenic mice
TMT: Tandem-mass-tag
W: Normal liver tissues of non-Tg
