# Supplementary figures and images for: Proteomic analysis revealed common, unique and systemic signatures in gender-dependent hepatocarcinogenesis
Source: Biol Sex Differ. 2020 Aug 13;11:46. doi: 10.1186/s13293-020-00316-5 (PMC7427087; doi:10.1186/s13293-020-00316-5)

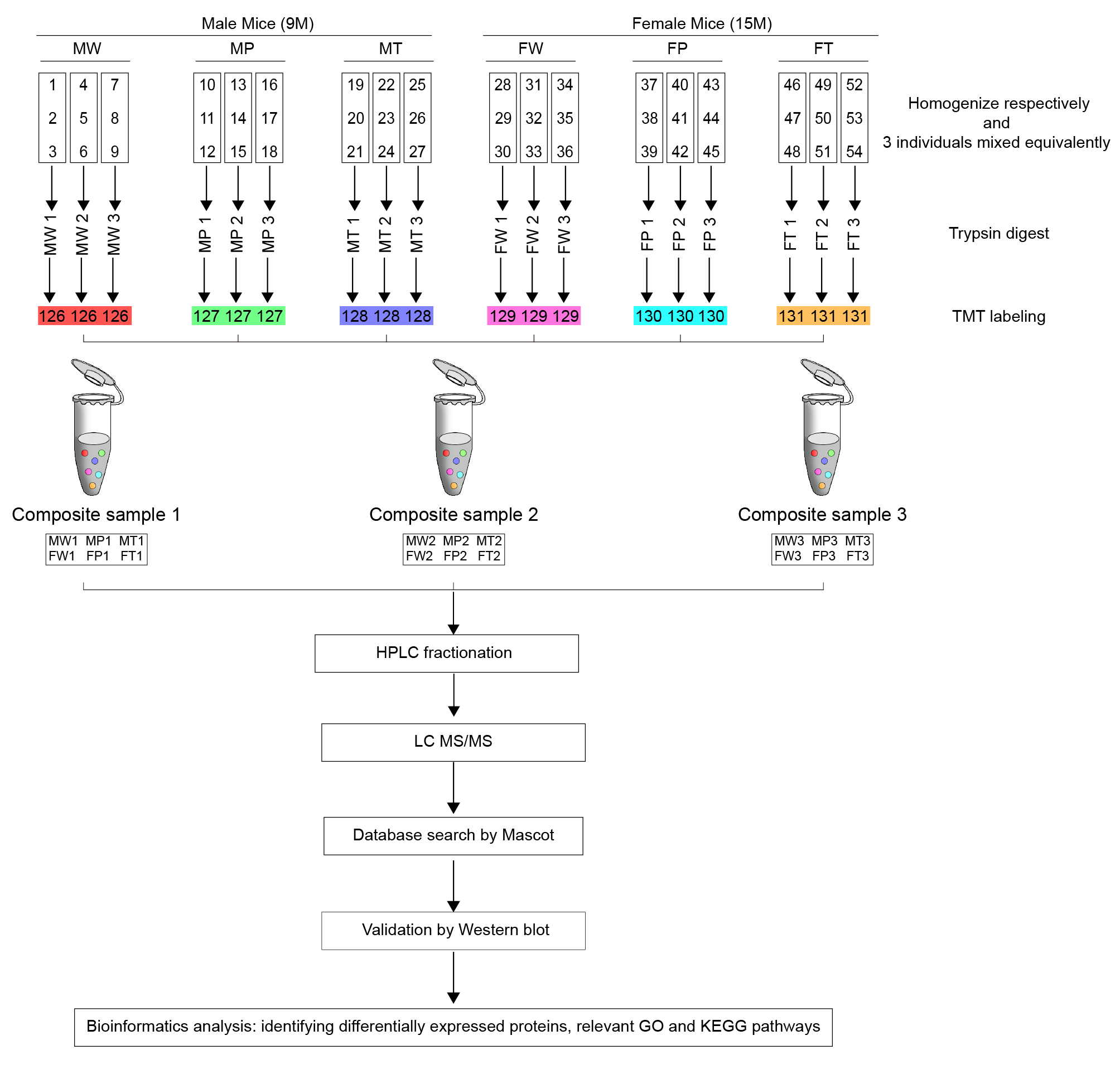

Supplement: Supplementary file 1 — Additional file 1: Figure S1. Experimental flowchart. Three sets of biologically replicate composite samples labeled with TMT (6-plex) were separated by HPLC and analyzed by LC-MS/MS. Proteins were identified using Maxquant software. After validation, the identified differentially expression proteins (DEPs) underwent bioinformatics analysis. MW, normal liver tissues of non-transgenic 9 months old males; MP, precancrous tissues of transgenic 9 months old males; MT, hepatocellular carcinoma tissues of transgenic 9 months old males; FW, normal liver tissues of non-transgenic 15 months old females; FP, precancerous tissues of transgenic 15 months old females; FT, hepatocellular carcinoma tissues of transgenic 15 months old females. The numbers indicate different individuals or different composite samples. [file 13293_2020_316_MOESM1_ESM.tif]

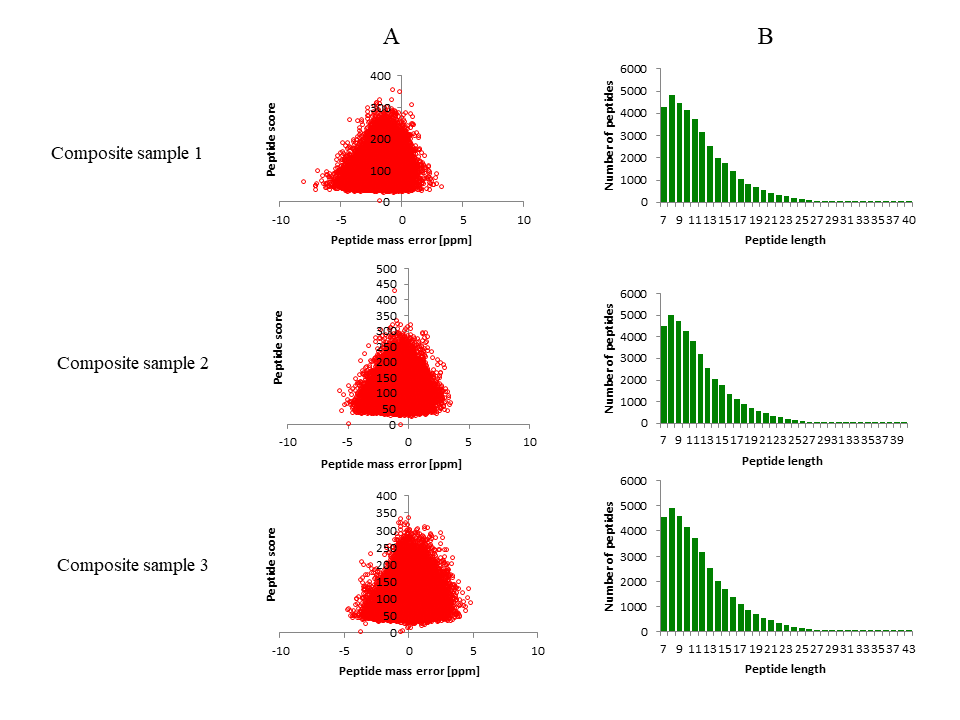

Supplement: Supplementary file 2 — Additional file 2: Figure S2. Depth of proteome coverage and quantitation for the three composite samples. (A) Mass offset distribution of peptides, (B) length distribution of peptides. [file 13293_2020_316_MOESM2_ESM.tif]

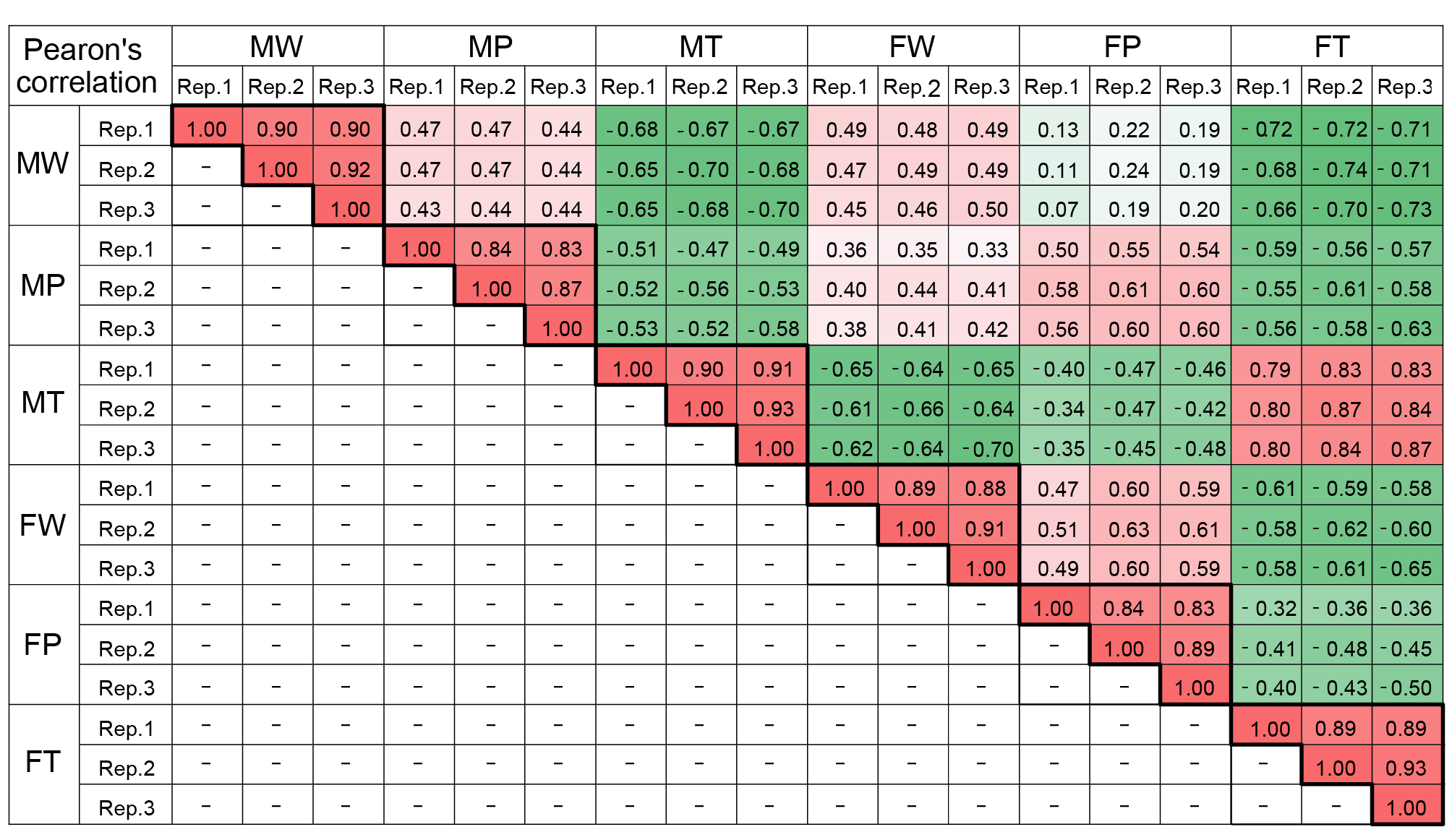

Supplement: Supplementary file 3 — Additional file 3: Figure S3. Heatmap of the Pearson’s correlation (R2) of the proteome dataset. [file 13293_2020_316_MOESM3_ESM.tif]

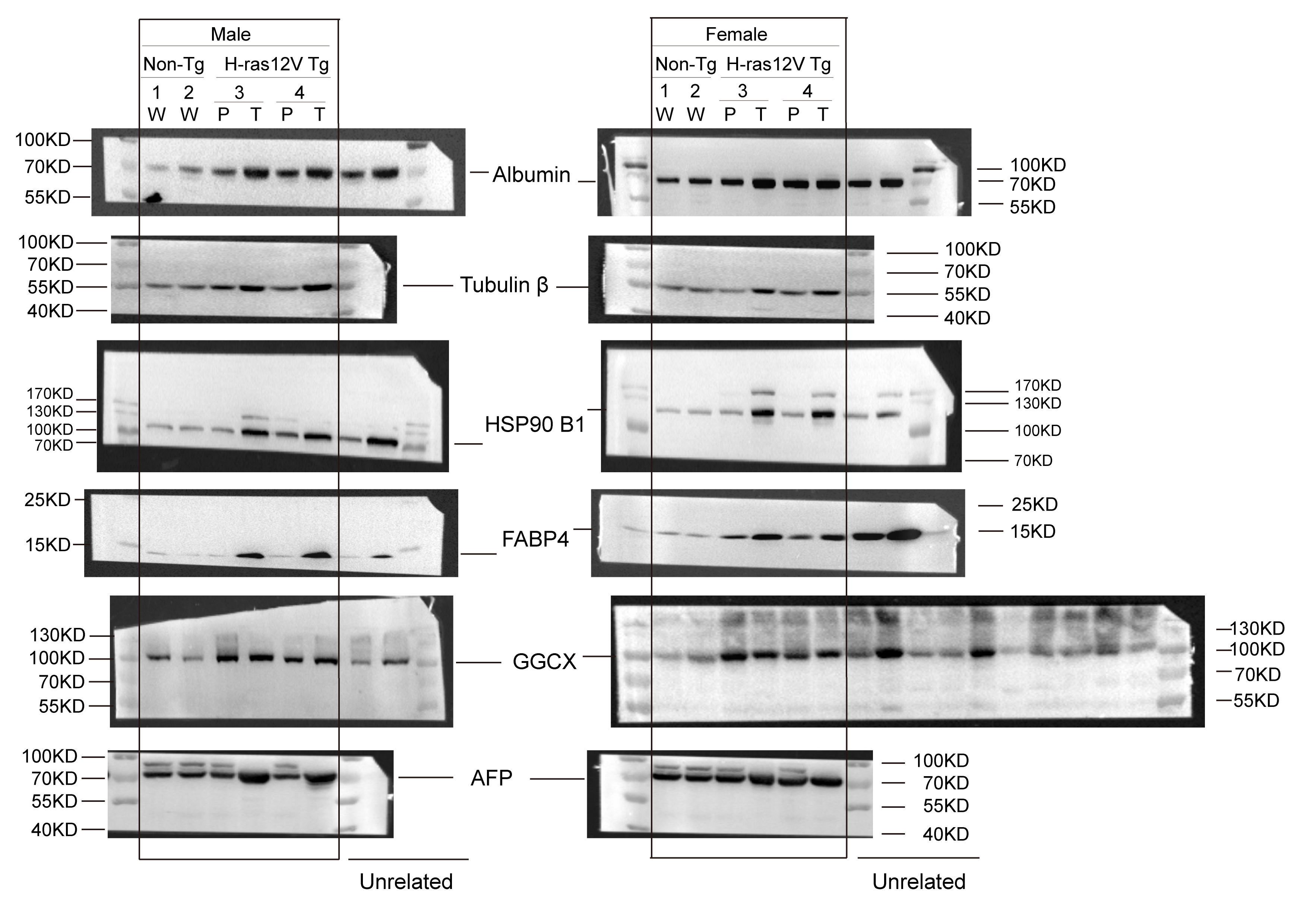

Supplement: Supplementary file 4 — Additional file 4: Figure S4. The presentation of images of original membrane for Western blot. [file 13293_2020_316_MOESM4_ESM.zip › Figure S4A.tif]

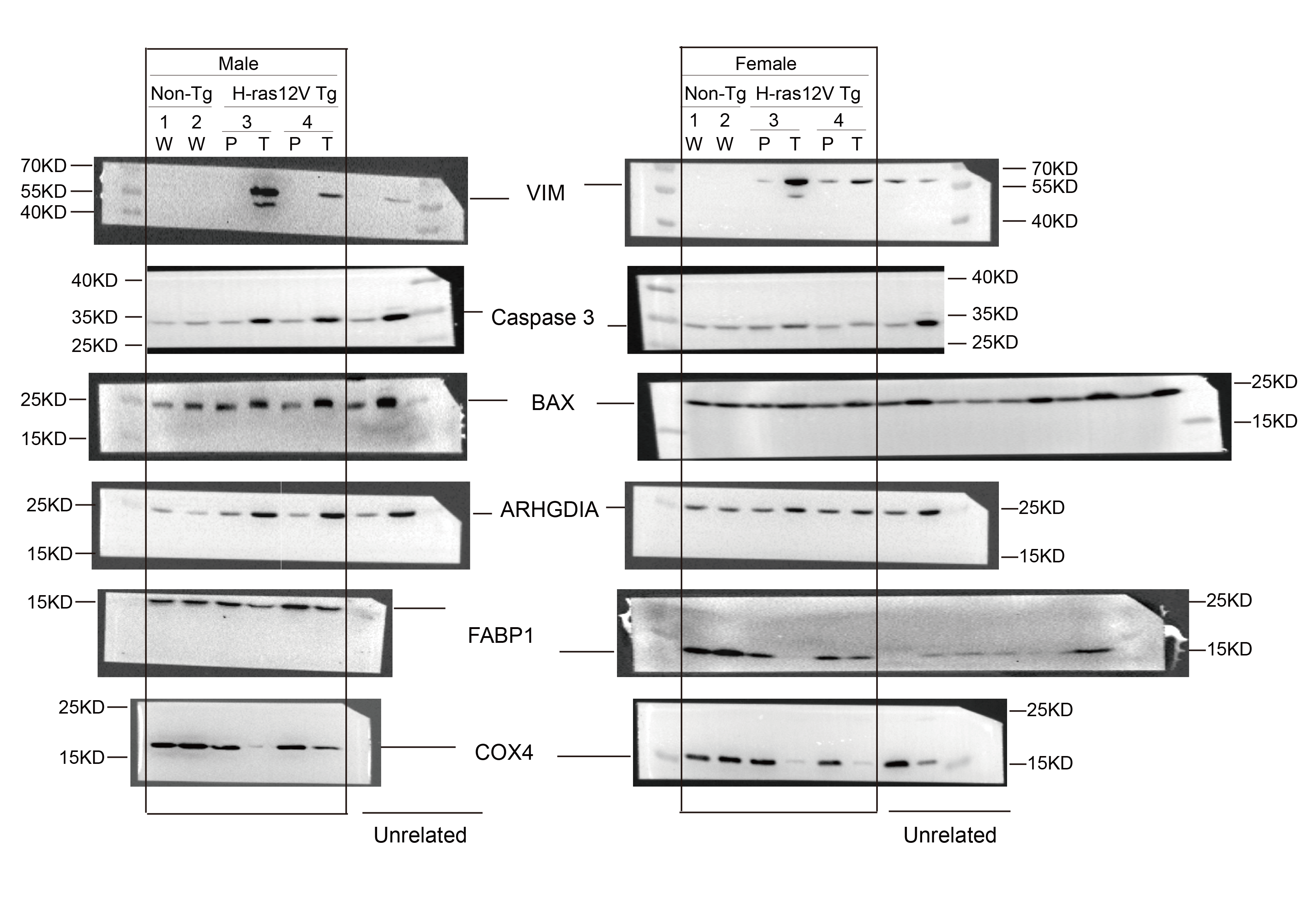

Supplement: Supplementary file 4 — Additional file 4: Figure S4. The presentation of images of original membrane for Western blot. [file 13293_2020_316_MOESM4_ESM.zip › Figure S4B.tif]

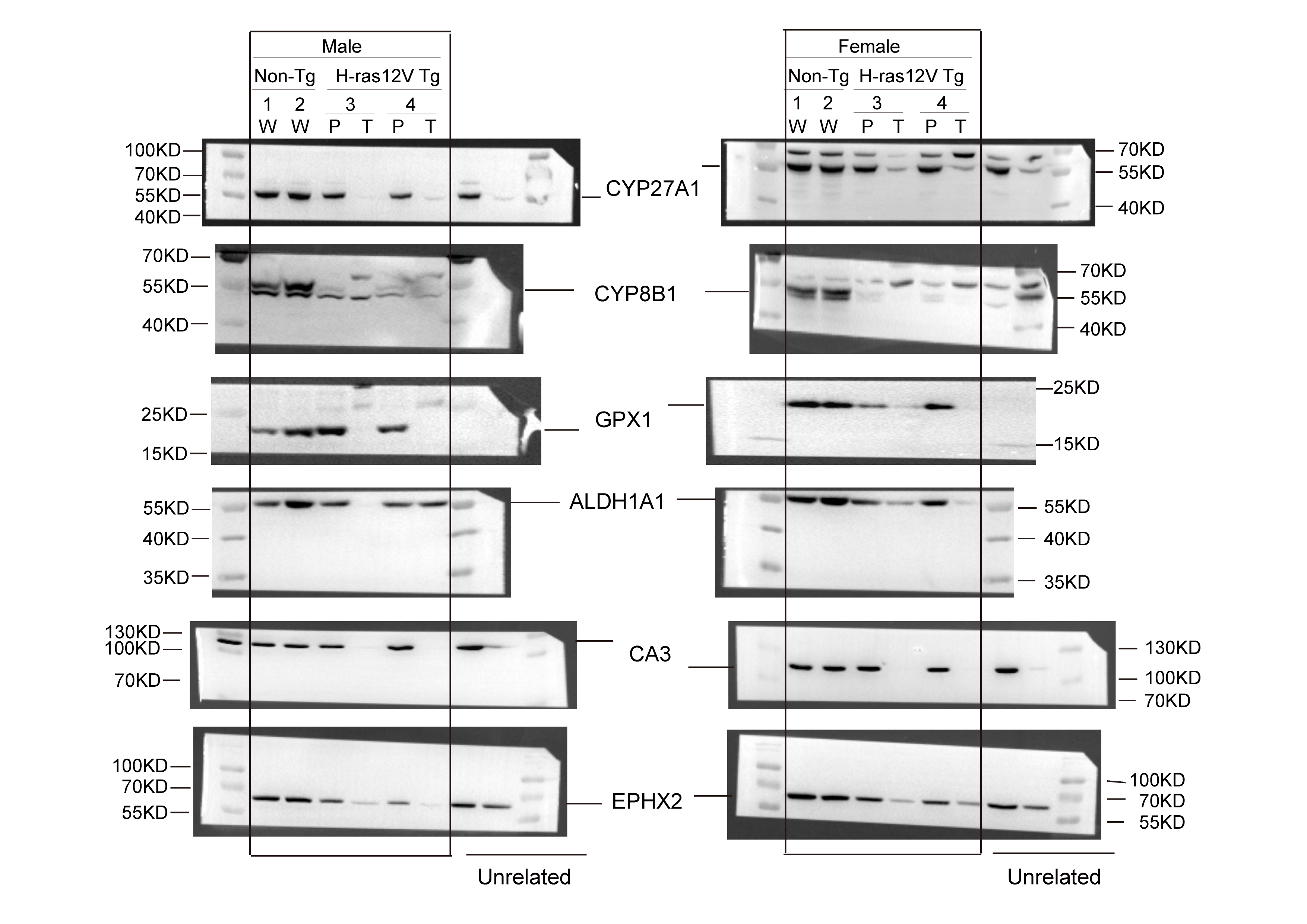

Supplement: Supplementary file 4 — Additional file 4: Figure S4. The presentation of images of original membrane for Western blot. [file 13293_2020_316_MOESM4_ESM.zip › Figure S4C.tif]

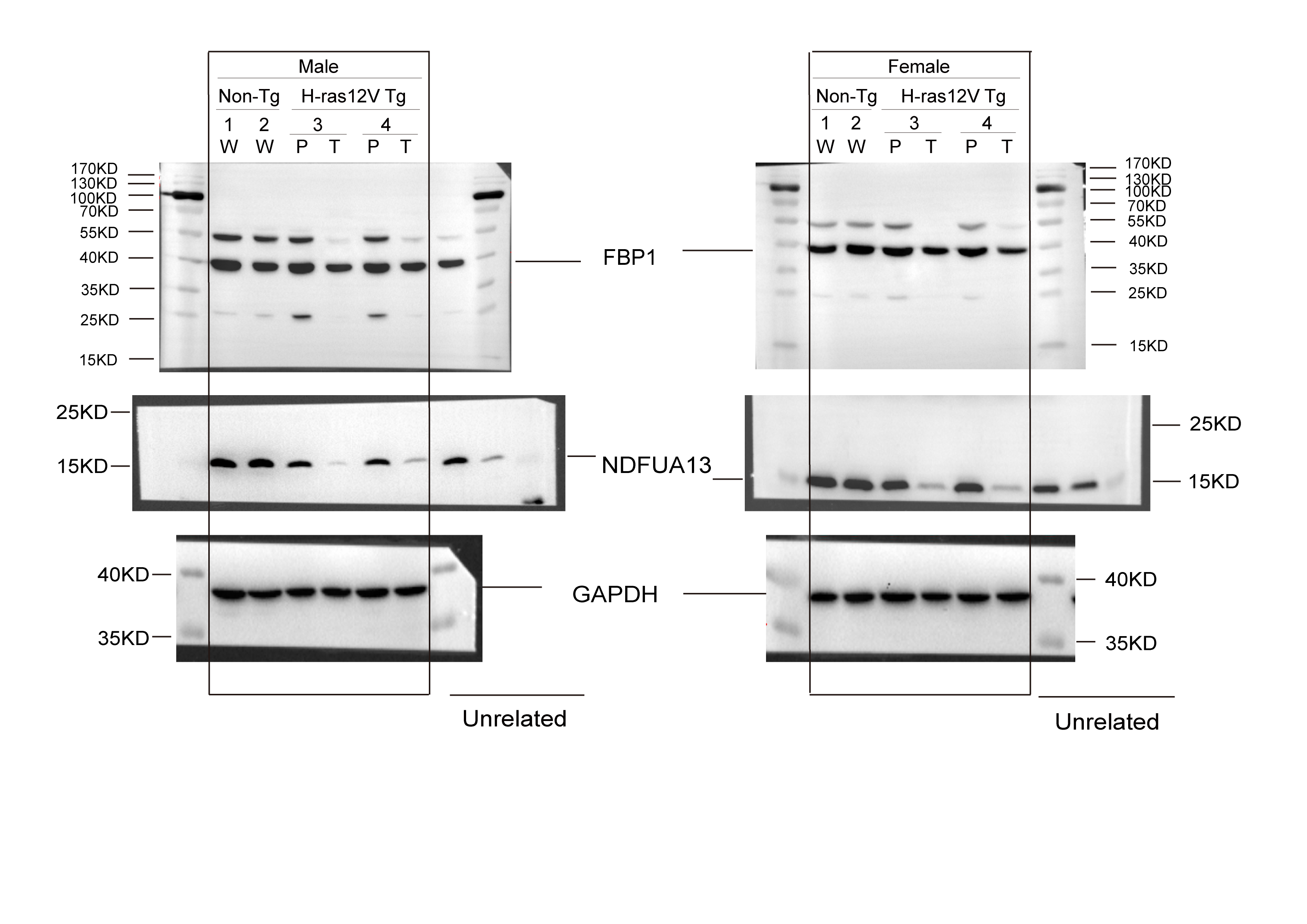

Supplement: Supplementary file 4 — Additional file 4: Figure S4. The presentation of images of original membrane for Western blot. [file 13293_2020_316_MOESM4_ESM.zip › Figure S4D.tif]

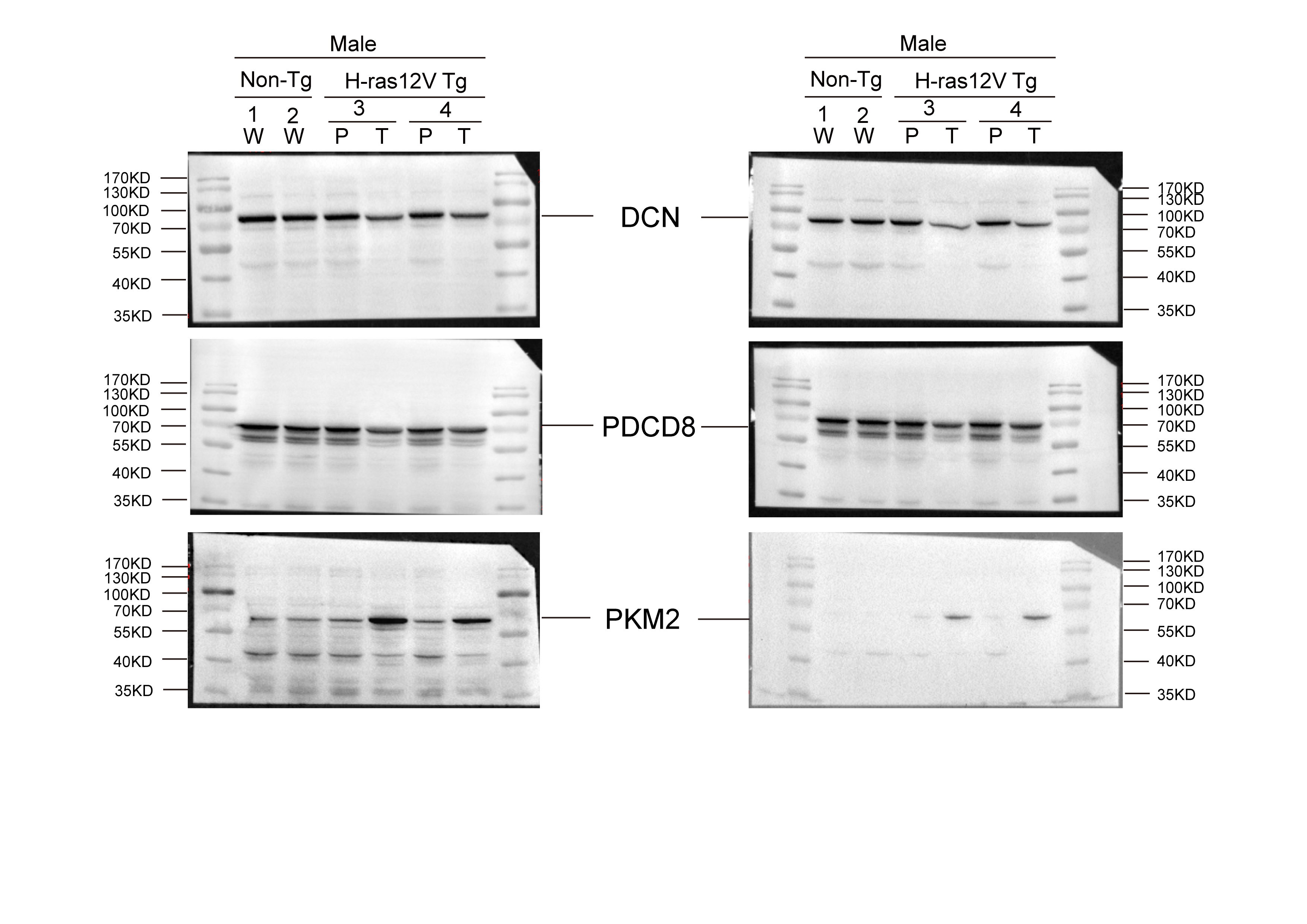

Supplement: Supplementary file 4 — Additional file 4: Figure S4. The presentation of images of original membrane for Western blot. [file 13293_2020_316_MOESM4_ESM.zip › Figure S4E.tif]

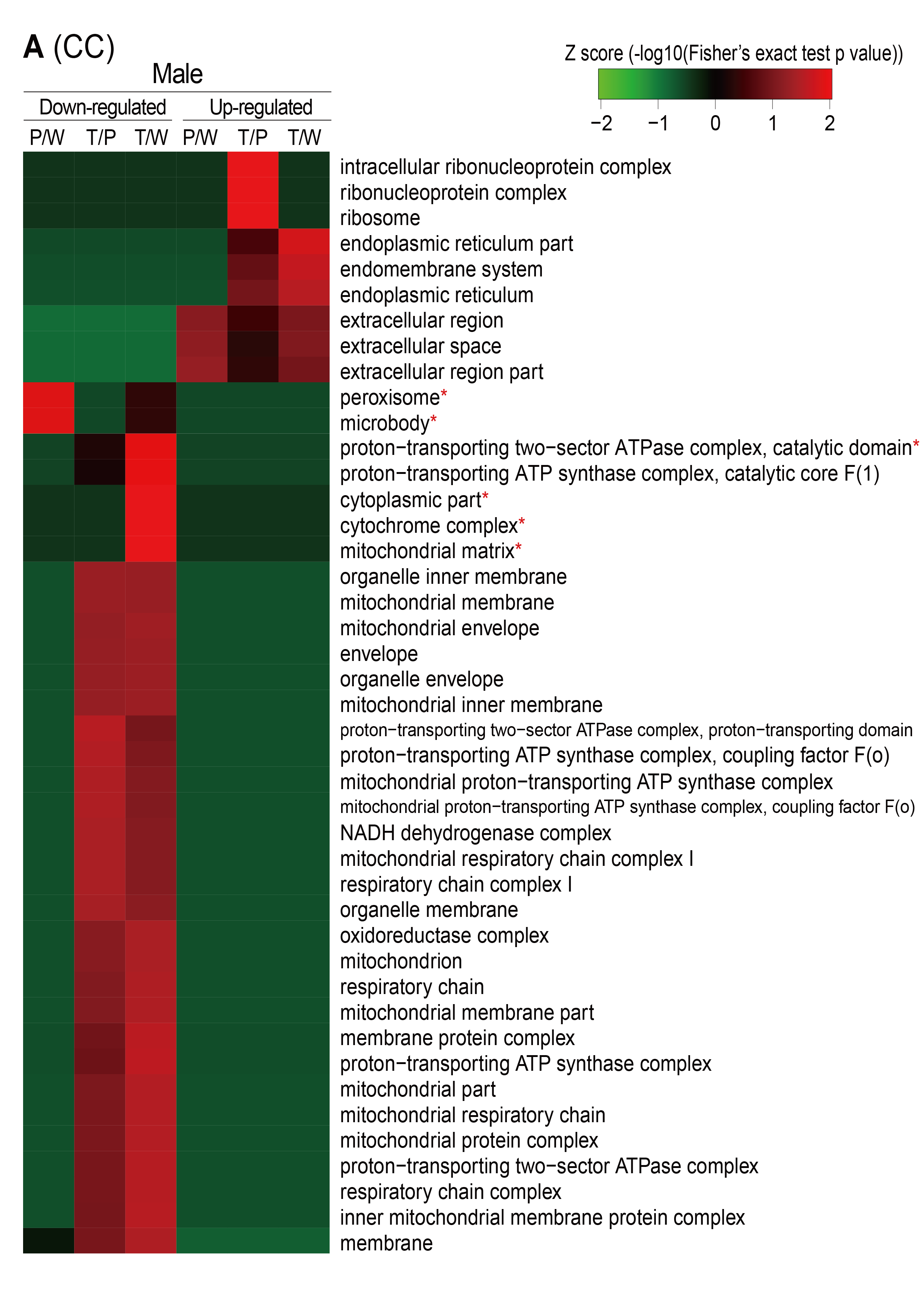

Supplement: Supplementary file 5 — Additional file 5: Figure S5. Bioinformatics analysis of DEPs during hepatocarcinogenesis in males and females. [file 13293_2020_316_MOESM5_ESM.zip › Figure S5A.tif]

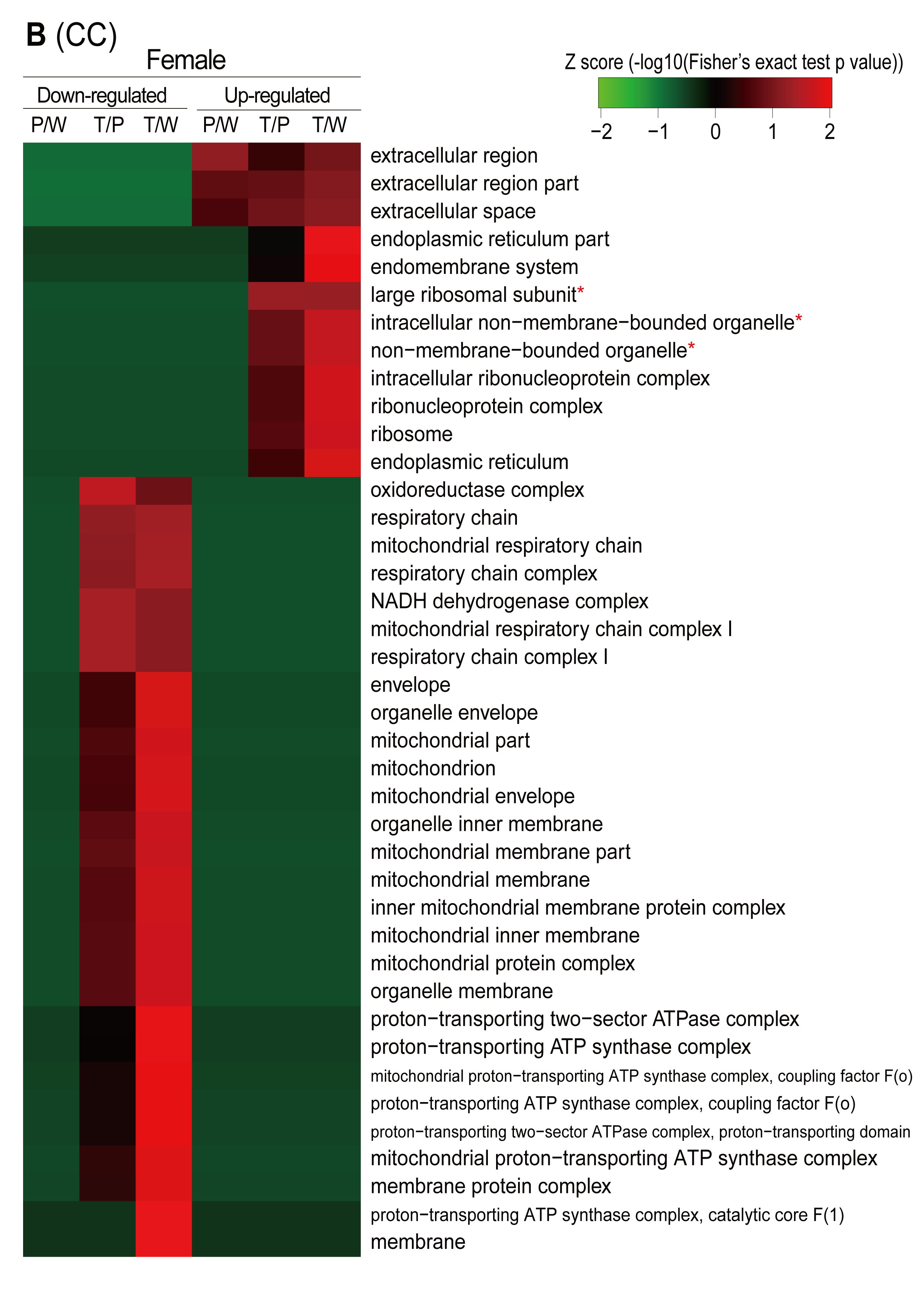

Supplement: Supplementary file 5 — Additional file 5: Figure S5. Bioinformatics analysis of DEPs during hepatocarcinogenesis in males and females. [file 13293_2020_316_MOESM5_ESM.zip › Figure S5B.tif]

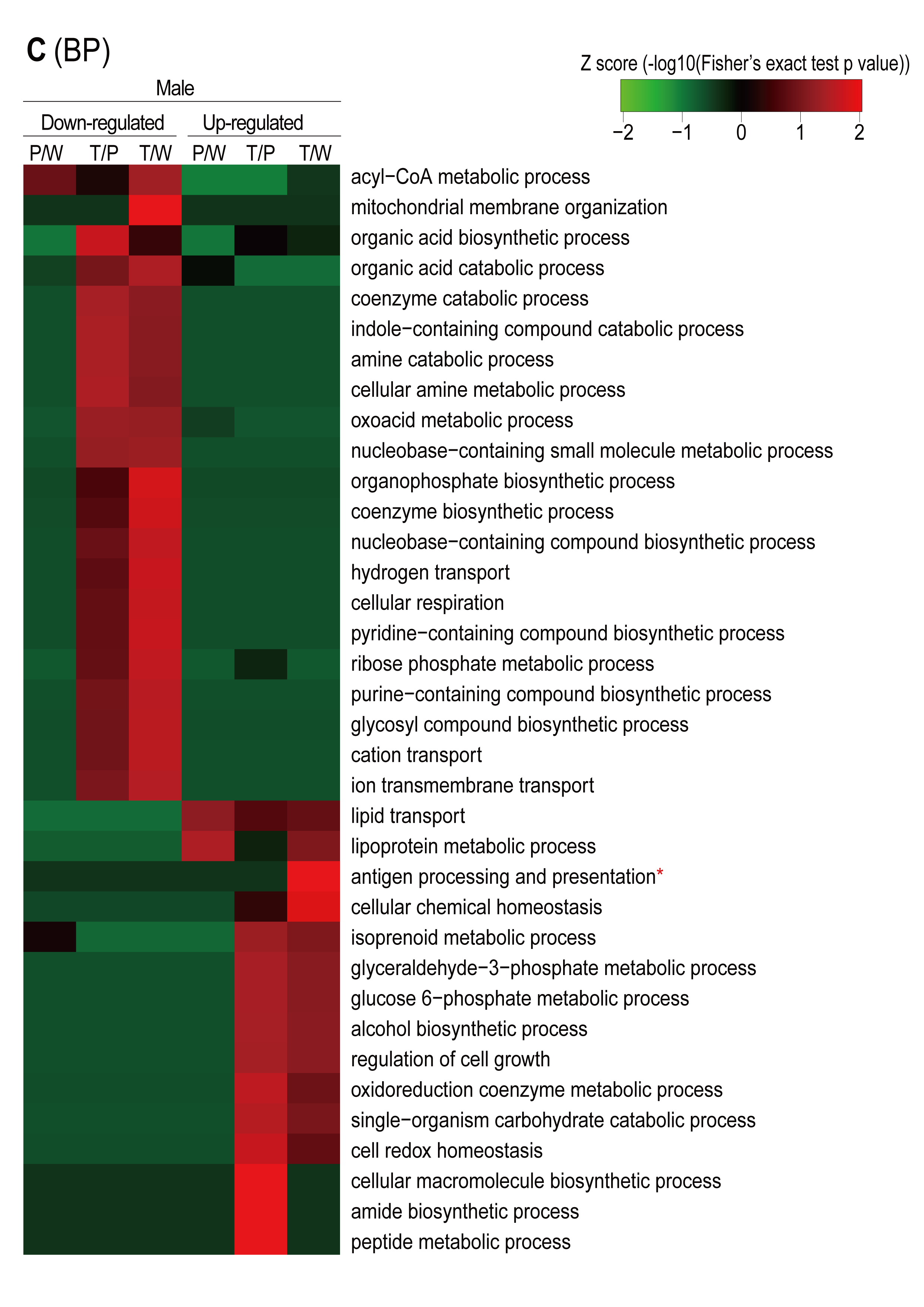

Supplement: Supplementary file 5 — Additional file 5: Figure S5. Bioinformatics analysis of DEPs during hepatocarcinogenesis in males and females. [file 13293_2020_316_MOESM5_ESM.zip › Figure S5C.tif]

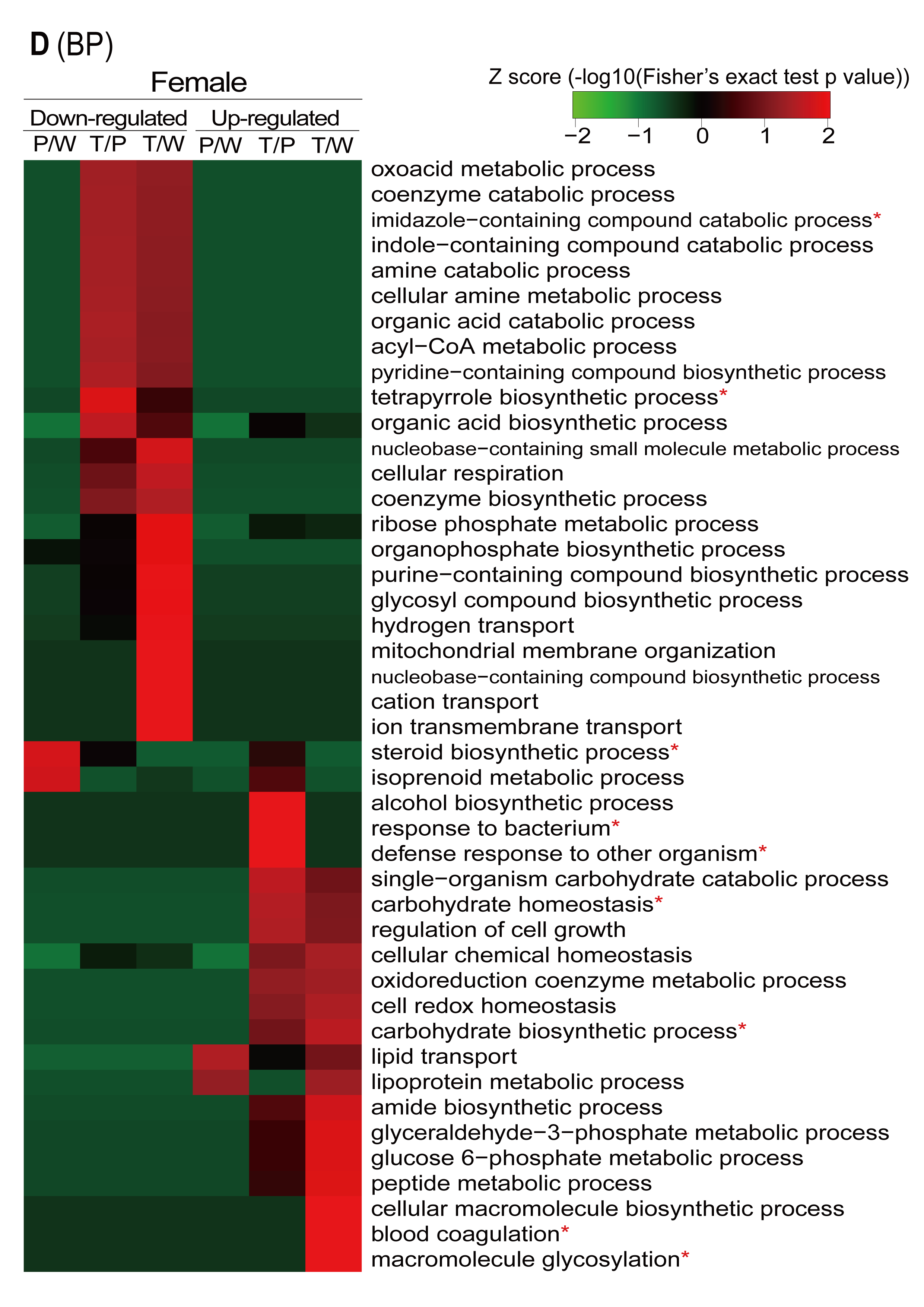

Supplement: Supplementary file 5 — Additional file 5: Figure S5. Bioinformatics analysis of DEPs during hepatocarcinogenesis in males and females. [file 13293_2020_316_MOESM5_ESM.zip › Figure S5D.tif]

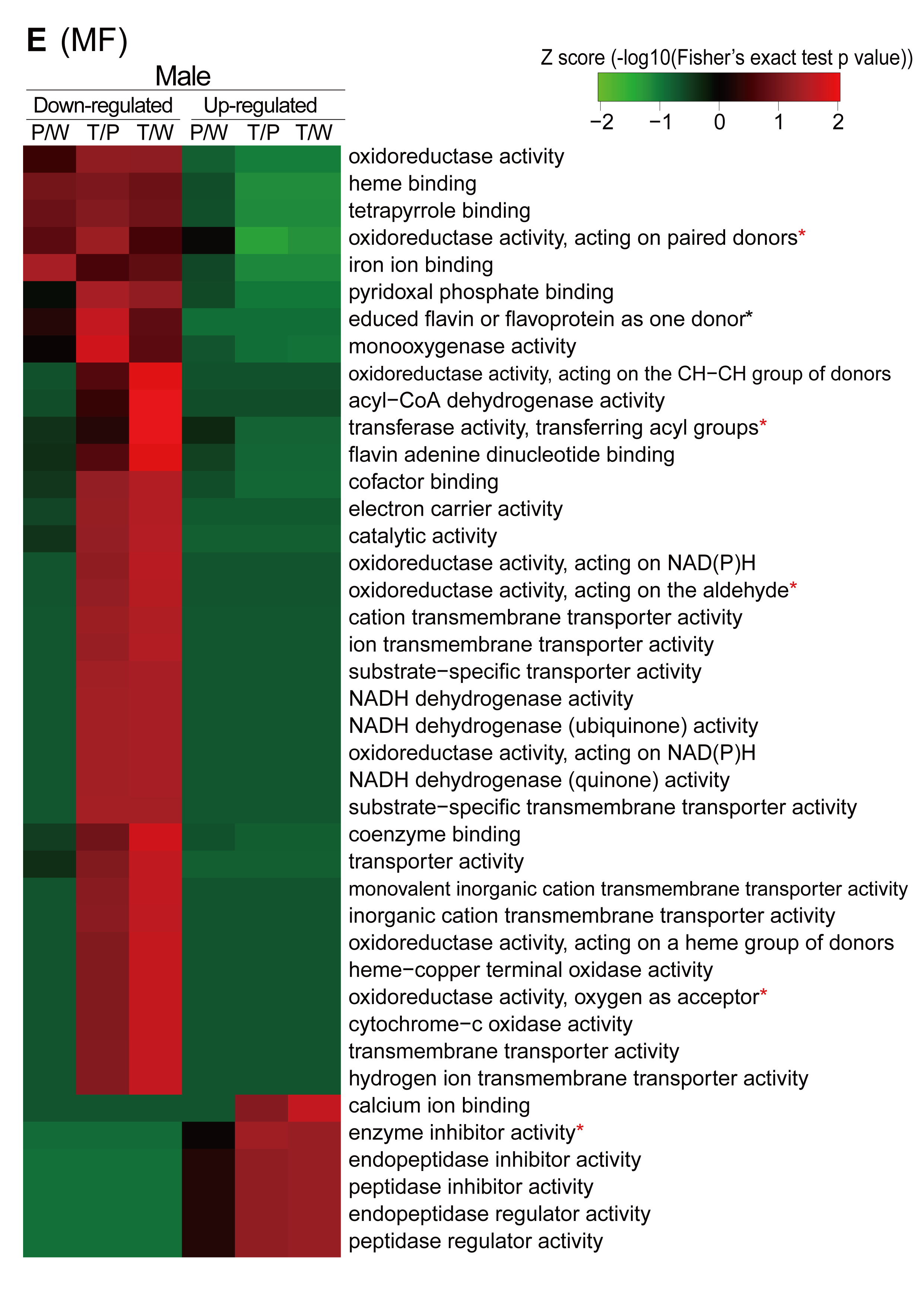

Supplement: Supplementary file 5 — Additional file 5: Figure S5. Bioinformatics analysis of DEPs during hepatocarcinogenesis in males and females. [file 13293_2020_316_MOESM5_ESM.zip › Figure S5E.tif]

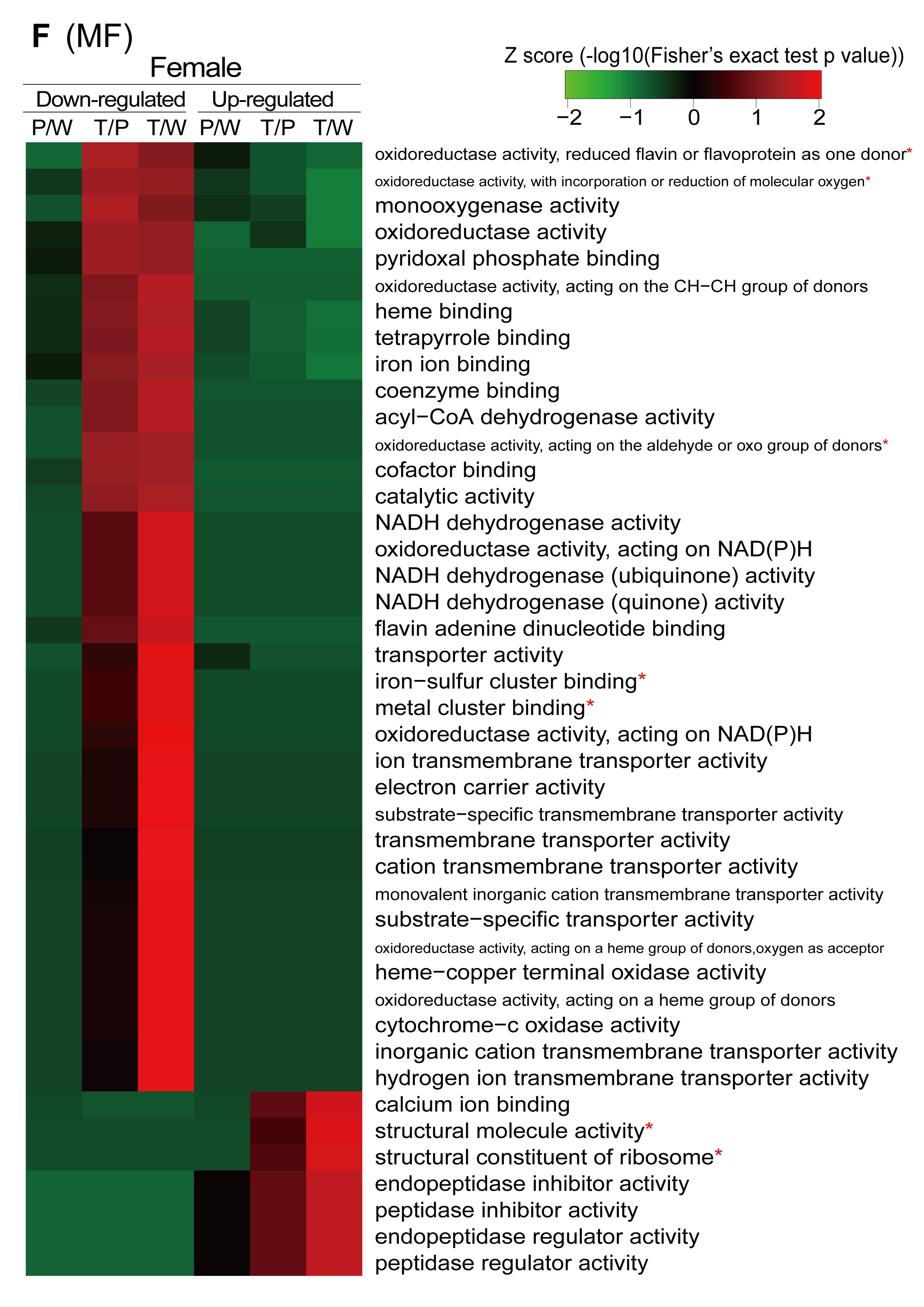

Supplement: Supplementary file 5 — Additional file 5: Figure S5. Bioinformatics analysis of DEPs during hepatocarcinogenesis in males and females. [file 13293_2020_316_MOESM5_ESM.zip › Figure S5F.tif]

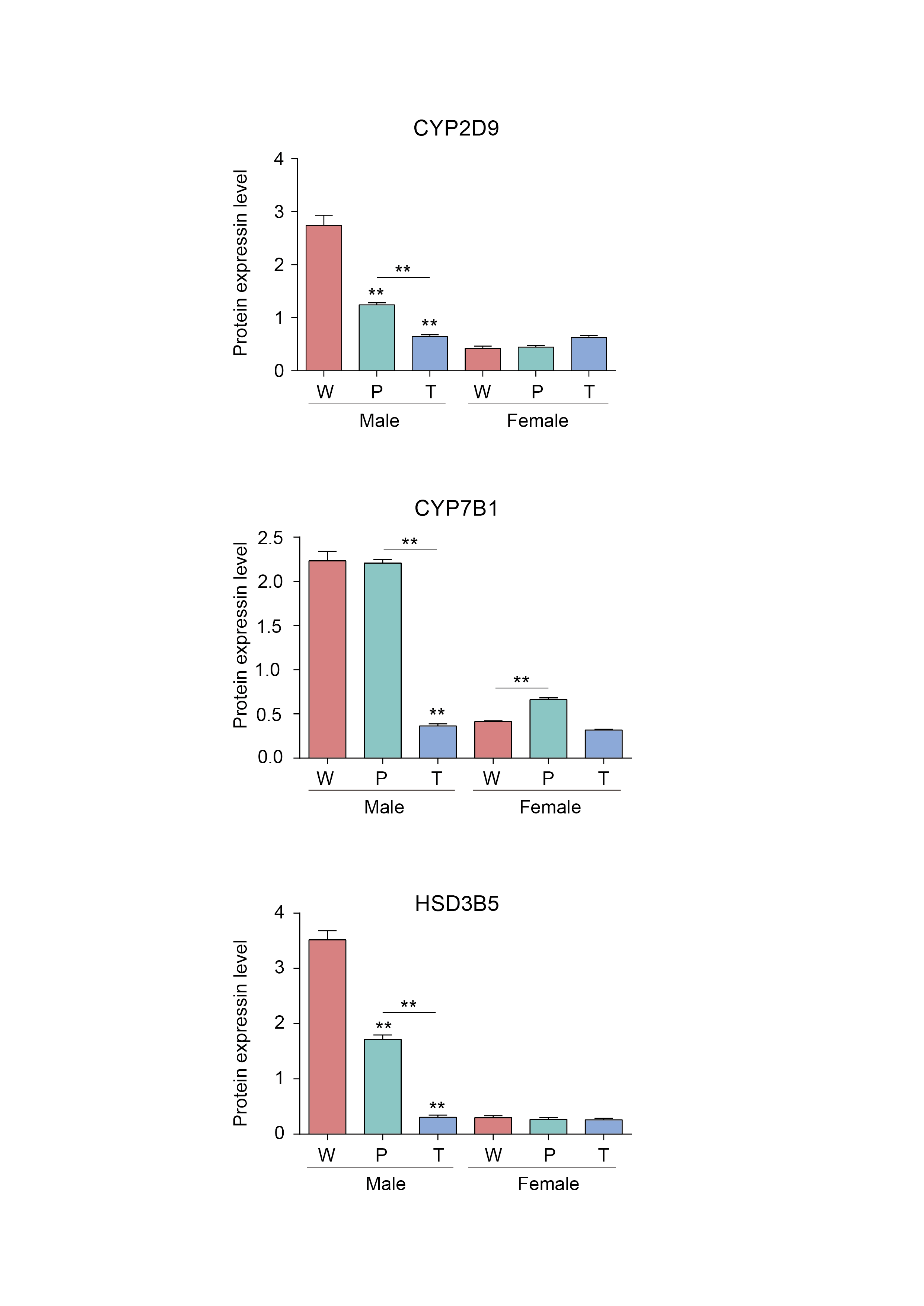

Supplement: Supplementary file 6 — Additional file 6: Figure S6. Expression levels of CYP2D9, CYP7B1, and HSD3B5 identified by TMT-based quantitative proteomic analysis. W, wild-type liver tissues; P, precancerous tissues of transgenic mice; T, hepatocellular carcinoma tissues of transgenic mice. The data are expressed as the mean ± SEM (n = 3) (**, p < 0.01). [file 13293_2020_316_MOESM6_ESM.tif]

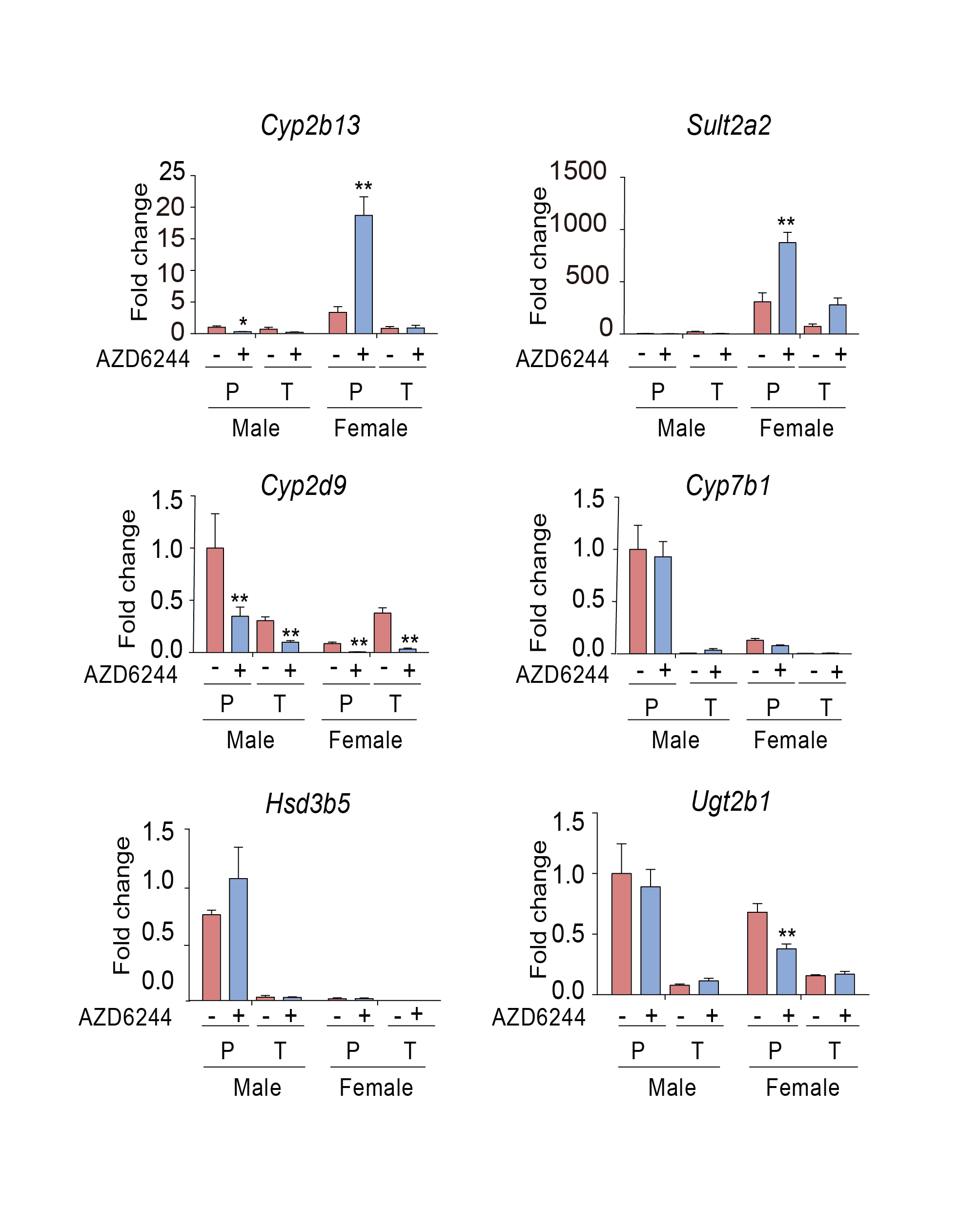

Supplement: Supplementary file 7 — Additional file 7: Figure S7. Changes in mRNA levels of Cyp2b13, Sult2a2, Cyp2d9, Cyp7b1, Hsd3b5, and Ugt2b1 in P and T treated by the ERK inhibitor AZD6244. P, precancerous tissues of transgenic mice; T, hepatocellular carcinoma tissues of transgenic mice. The mRNA levels of genes were detected by RT-qPCR and normalized to Rp135a. The data are expressed as the mean ± SEM (n = 5-6). (*, p < 0.05; **, p < 0.01). [file 13293_2020_316_MOESM7_ESM.tif]
