# Supplementary material for: Proteomic analysis revealed common, unique and systemic signatures in gender-dependent hepatocarcinogenesis
Source: Biol Sex Differ. 2020 Aug 13;11:46. doi: 10.1186/s13293-020-00316-5 (PMC7427087; doi:10.1186/s13293-020-00316-5)
Supplement: Supplementary file 17 — Additional file 17: Table S10. Primer sequences for RT-qPCR. [file 13293_2020_316_MOESM17_ESM.docx]

| Genes | Forward(5’to3’) | Reverse(5’to3’) |
| --- | --- | --- |
| *Ar* | CGCTTCTACCAGCTCACCAA | TCAGGAAAGTCCACGCTCAC |
| *Esr1* | TCTGACAATCGACGCCAGAAT | GCATAGTCATTGCACACGGC |
| *Cyp2b13* | CCGGCTACCAACCCTTGATG | CTTTGGTCACTCTGTGTGGTG |
| *Sult2a2* | CCGAGAGATATTCTTGTGTCTGGT | GTCCCCAGTTGTACCATTTCCT |
| *Cyp2d9* | CGCATGGAGCTTTTCCTCTTC | AGGGGGCAACAAGAATACCAT |
| *Cyp7b1* | CGGCATCCTGAAGCTATGGAA | CAAGCTGTCCAATTGTTCTCTGG |
| *Ugt2b1* | CAGCTCTGCATGACCTTACCT | TGGCAACAAAAGAGGCAACATT |
| *Hsd3b5* | TTCCTGCTACGTCCAGTTTACA | TGGCTGATAGCCTAGATCGC |
| *Rp135a*  *Cyp3a41* | CCGAGATGAAACGGAGTTCTAC  CTTTGTGGAGAAAGCCAAAGGG | GTTACCTTTCCCCAGATCACT  TGAATCCTTTGGGAACATGCAGA |

**Table S10.** Primer sequences for RT-qPCR.
